# Foundry-compatible high-resolution patterning of vertically phase-separated semiconducting films for ultraflexible organic electronics

**DOI:** 10.1038/s41467-021-25059-8

**Published:** 2021-08-16

**Authors:** Binghao Wang, Wei Huang, Sunghoon Lee, Lizhen Huang, Zhi Wang, Yao Chen, Zhihua Chen, Liang-Wen Feng, Gang Wang, Tomoyuki Yokota, Takao Someya, Tobin J. Marks, Antonio Facchetti

**Affiliations:** 1 Joint International Research Laboratory of Information Display and Visualization, Key Laboratory of MEMS of Ministry of Education, School of Electronic Science and Engineering, Southeast University, Nanjing, Jiangsu PR China; 2 Department of Chemistry and the Materials Research Center, Northwestern University, Evanston, IL USA; 3 Department of Electrical Engineering and Information Systems, School of Engineering, The University of Tokyo, Bunkyo-ku, Tokyo Japan; 4 Institute of Functional Nano & Soft Materials (FUNSOM), Jiangsu Key Laboratory for Carbon Based Functional Materials and Devices, Soochow University, Suzhou, Jiangsu PR China; 5 Research Center for Engineering Technology of Polymeric Composites of Shanxi Province, School of Materials Science and Engineering, North University of China, Taiyuan, Shanxi PR China; 6 Flexterra Inc., Skokie, IL USA; 7 College of Chemistry, Sichuan University, Chengdu, Sichuan PR China; 8 State Key Laboratory for Modification of Chemical Fibers and Polymer Materials, International Joint Laboratory for Advanced Fiber and Low-Dimension Materials, College of Materials Science and Engineering, Donghua University, Shanghai, PR China

**Keywords:** Electrical and electronic engineering, Materials for devices

## Abstract

Solution processability of polymer semiconductors becomes an unfavorable factor during the fabrication of pixelated films since the underlying layer is vulnerable to subsequent solvent exposure. A foundry-compatible patterning process must meet requirements including high-throughput and high-resolution patternability, broad generality, ambient processability, environmentally benign solvents, and, minimal device performance degradation. However, known methodologies can only meet very few of these requirements. Here, a facile photolithographic approach is demonstrated for foundry-compatible high-resolution patterning of known p- and n-type semiconducting polymers. This process involves crosslinking a vertically phase-separated blend of the semiconducting polymer and a UV photocurable additive, and enables ambient processable photopatterning at resolutions as high as 0.5 μm in only three steps with environmentally benign solvents. The patterned semiconducting films can be integrated into thin-film transistors having excellent transport characteristics, low off-currents, and high thermal (up to 175 °C) and chemical (24 h immersion in chloroform) stability. Moreover, these patterned organic structures can also be integrated on 1.5 μm-thick parylene substrates to yield highly flexible (1 mm radius) and mechanically robust (5,000 bending cycles) thin-film transistors.

## Abstract (web summary)

Though shape-changing devices are promising for future haptic displays, existing designs fail to provide smooth surfaces for the user during tactile exploration. Here, the authors utilize flexible auxetic structures to realize shape displays with smooth surfaces and different Gaussian curvatures.

## Introduction

To minimize electronic device feature sizes, eliminate crosstalk in circuitry, and scale-up soft matter opto-electronic device fabrication, foundry-compatible patterning of all functional layers is essential for creating multiple circuitry layers, and systems integration^1–6^. Specifically, high-resolution patterning of robust semiconductor films in thin-film transistor (TFT) arrays must optimize the charge transport and on-current/off-current ratio (*I*_on_:*I*_off_) ratio, while achieving reliable deposition by solution-processing of all additional non-TFT components, including the gate dielectric/gate contact in top-gated TFTs, and the source-drain electrodes in top-contact TFTs, as well as planarization/passivation layers in both architectures^7–12^. Several pioneering studies realized patternable photo­crosslinked polymer semiconductors by appending crosslinkable moieties to the polymer backbone. Following these approaches, crosslinked patterned films of various polythiophenes with 50–100 µm features and TFT mobilities of 10^−3^–10^−1^ cm^2^ V^−1^ s^−1^ were demonstrated^13–16^. To further enhance pattern resolution, alternative approaches have focused on developing chemically orthogonal photoresist/protective layers to preserve the underlying semiconductor layer integrity during photolithography^1,8,17,18^. In this way high-resolution features (1–5 µm) were realized with mobilities of ca. 10^−1^ cm^2^ V^−1^ s^−1^. Nevertheless, foundry-compatible patterning methodologies that are low-cost, ambient processable, environmentally benign, highly efficient and reliable, and enable high-resolution patterned features without compromising device performance have remained elusive. Thus, the existing patternable semiconductors for orthogonal photolithography are chemically and/or morphologically unstable in most solvents as well as metal etchants, and to date cannot be realistically implemented in circuitry fabrication. Moreover, the crosslinking of functionalized polymeric semiconductors typically relies on high deep-UV radiation doses in inert atmosphere, which is incompatible with efficient FAB processing.

**Fig. 1:**
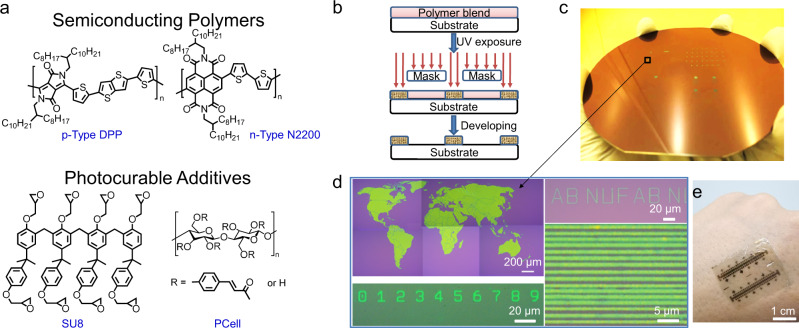
Materials and methodology used to produce high-resolution patterned ultraflexible soft matter electronic circuitry. **a** Chemical structures of the semiconductors and photocurable additives used in this study. **b** Schematic of the photolithographic process. **c** Photograph of a patterned 50% DPP/SU8 (DPP:SU8 = 1:1 w/w) film on a 4-inch SiO_2_/Si wafer. **d** Optical images of 50% DPP/SU8 films with various patterned topologies. **e** Photographic image of ultraflexible 50% DPP/SU8-based organic TFTs (OTFTs) on a human hand.

Here we report a foundry-compatible wafer-scale patterning approach for creating polymeric semiconducting layers with fidelity at the 0.5 μm resolution scale. The robust patterned semiconducting layers are produced from a phase-separated blend of readily available p- or n-type polymeric semiconductors and a compatible photo-crosslinkable additive (Fig. 1a). The patterned layers are chemically inert to aggressive aqueous and organic solvents, and thereby withstand subsequent deposition and patterning of the additional organic layers and/or metal contacts used in the circuitry fabrication. To illustrate the generality of this methodology and availability of all components, we employ both p-type, poly[2,5-bis(2-octyldodecyl)-3,6-diketopyrrolopyrrole-alt-5,5’-(2,5-di(thien-2-yl)thieno[3,2-*b*]thiophene)] (DPP) and n-type, poly[[*N, N*-bis (2-octyldodecyl)-napthalene-1, 4, 5, 8-bis (dicarboximide)-2, 6-diyl]-*alt*-5, 5’-(2,2’-bithiophene)] (N2200) semiconducting polymers paired with photocurable additives (PAs) such as commercially available SU-8 2000.5 (abbreviated SU8) and a Northwestern cinnamate-functionalized cellulose polymer abbreviated PCell)^19^. The vertical phase separation (VPS) and semiconductor nanofiber morphology in the resulting blend films are confirmed by comprehensive characterization techniques including atomic force microscopy (AFM), time-of-flight secondary ion mass spectrometry (ToF-SIMS), and 2D grazing incidence wide angle X-ray scattering (GIWAXS). The patterning process is highly efficient and minimizes waste since it requires only 3 steps, versus 7+ steps for conventional photolithography (Fig. 1b and Supplementary Fig. 1). Furthermore, both the film deposition and patterning processes can be effectively carried out using environmentally benign green solvents such as tetrahydrofuran (THF) as a proof-of-concept. The fabricated OTFTs have carrier mobilities of 0.1–0.24 cm^2^ V^−1^ s^−1^, comparable to those of the pristine polymers, but with far more efficient switching (subthreshold swing = 1.4 V dec^−1^), greater thermal stability (175 °C), and superior mechanical durability (5000 bending cycles at a radius of 1 mm).

## Results

### Semiconductor blend film and device fabrication

**Fig. 2:**
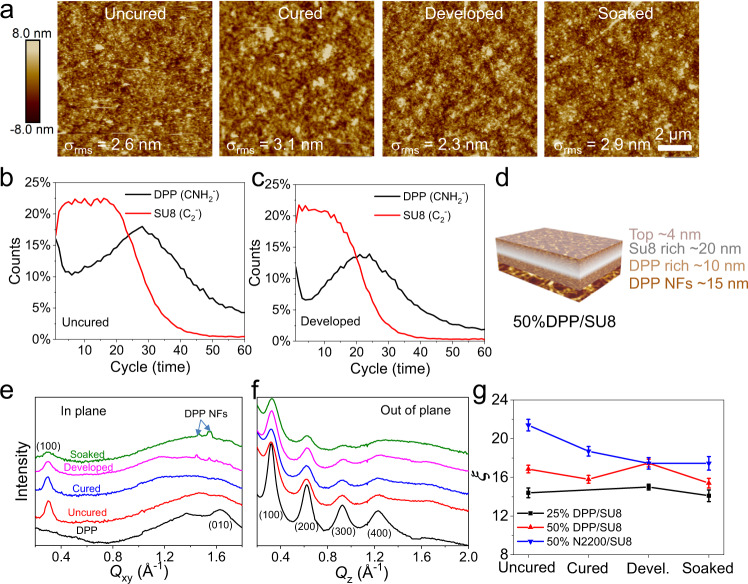
Characterization of materials phase separation processes enabling transistor fabrication for high-resolution patterned ultraflexible soft matter electronic circuitry. **a** AFM images of a 50% DPP/SU8 films at different stages of the photolithographic process, demonstrating excellent chemical resistance. The ToF-SIMS depth profiles for **b** uncured and **c** developed 50% DPP/SU8 films. **d** Schematic of VPS in uncured 50% DPP/SU8 films into SU-8 rich, DPP-rich, and DPP nanofibers, based on the AFM and ToF-SIMS characterization. **e** In-plane and **f** out-of-plane GIWAXS line cuts of the indicated 50% DPP/SU8 films. **g** Coherence lengths (ξ) derived from out-of-plane (200) reflections for DPP/SU8 and in-plane (100) reflections for 50% N2200/SU-8 after the indicated processing conditions. The error bars provide the standard deviation.

The semiconducting polymers DPP and N2200 were selected because they are among the most efficient p- and n-type semiconductors for OTFTs while SU8 and PCell are inexpensive and readily available. Regarding the OTFT architectures, and to demonstrate architecture generality, we preferentially investigated bottom-gate top-contact (BGTC) OTFTs for DPP and top-gate bottom-contact (TGBC) OTFTs for N2200 since they are those where the pristine polymers perform the best. Details of the semiconductor blend film deposition, curing/patterning, and OTFT fabrication processes are reported in the Supporting Information. Briefly, in a typical experiment for bottom-gate top-contact (BGTC) OTFT fabrication, used for the p-type DPP-based devices, the semiconductor/PA solution (x% in weight semiconductor vs. PA, x = 25, 50, and 75; total concentration = 4 mg mL^−1^) was spin-coated on trichloro(octyl)silane-treated SiO_2_/Si substrates at 1500 rpm for 30 s in ambient with ~30% relative humidity. After baking the films at 95 °C for 1 min, they were exposed through a shadow mask to a 365 nm ultraviolet (UV) light (Electrodeless UV lamp, dose = 60–80 mJ cm^−^^2^) or by a maskless aligner for high-resolution patterning, followed by annealing at 95 °C for 2 min (Fig. 2b). Next, the resulting cured x% DPP/SU8 blend films were developed with CHCl_3_ for 5 s, to achieve the desired patterned films with thickness of 40–70 nm, depending on the semiconductor and semiconductor/PA weight ratio. The BGTC OTFT structures were completed by thermally depositing gold source/drain contacts with channel width/length (*W/L*, μm) of 1000/100 or 40/10, defined by metal mask or lift-off process, respectively (*vide infra*). Top-gate bottom-contact (TGBC) OTFTs were employed for n-type N2200-based devices, which were fabricated/patterned on glass substrates with thermally evaporated Au source-drain contact (*W/L* = 1000/100) and completed by spin-coating a 740 ± 10 nm-thick poly(methyl methacrylate) (PMMA) gate dielectric layer (*C*_i_ = 3.6 nF cm^−2^), and thermally evaporating a gold gate contact. Fig. 1c, d, and Supplementary Fig. 2 show representative optical images of photo-patterned DPP:SU8 films, demonstrating excellent reproducibility and reliability over a large scale for patterns of different shapes and feature sizes. The minimum features achievable are ~0.5 µm, limited by the maskless aligner (Heidelberg MLA150), and the calculated line width roughness (LWR) and line edge roughness (LER) are 97.8 ± 1.5 nm and 70.1 ± 1.2 nm, respectively. These values are superior to those for conventional several micrometer-thick photoresists (Supplementary Figs. 3 and 4). The generality of this three-step patterning process was verified with other blends such as DPP/PCell, N2200/SU8, and N2200/PCell (Supplementary Figs. 4–8) on glass or ultraflexible polymer substrates (Fig. 1e).

### Blend film morphological characterization

The film morphologies and microstructures of the semiconductor/PA films during the patterning process were investigated by AFM, ToF-SIMS, and GIWAXS. Fig. 2a and Supplementary Fig. 9 show representative AFM images of pristine DPP and 50% DPP/SU8 (DPP:SU8 = 1:1 w/w) films before photocuring (uncured), after UV irradiation (cured), after development (developed/patterned), and after 24 h immersion in CHCl_3_ (soaked). The pristine DPP films (120 nm thick) exhibit uniform crystalline domain structures with an rms roughness (σ_rms_) = 1.7 nm, while the 50% DPP/SU8 film (~50 nm thick) has smaller crystalline domains and a slightly rougher surface (σ_rms_ = 2.6 nm). Negligible morphological and thickness changes are evident for 50% DPP/SU8 films after UV irradiation. After developing, the film thickness is found to contract slightly to ~45 nm due to partial removal of the top DPP/SU8 portion. Impressively, negligible morphological and thickness changes are observed on 24 h immersion in CHCl_3_, retaining a σ_rms_ = 2.9 nm. This blend film thickness and morphology evolution with curing/developing indicate multilayer VPS in the semiconductor/polymer blends as supported by the ToF-SIMS data (Fig. 2b–d and Supplementary Fig. 10). The C_2_^−^ and CNH^−^ ion signals are signatures of SU8 and the DPP, respectively, and their ratio can be used to assay the distribution of the two components with film depth.^25^ Thus, the ToF-SIMS depth profile for uncured 50% DPP/SU8 samples (1 etching cycle represents ~1 nm, Fig. 2b) indicates formation of a multilayer structure (Fig. 2d) with a top layer having comparable SU8-DPP contents and of thickness ~4 nm, a central layer greatly enriched in SU8 with thickness of ~20 nm, and a lower layer primarily consisting of DPP (~10 nm), and then exclusively (another ~15 nm), composed of DPP nanofibers (NFs).^20–26^ Regarding the developed 50% DPP/SU8 sample, which is only ~5 nm thinner than the undeveloped sample, it’s ToF-SIMS result indicates a similar composition profile with only the film upper portion affected. However, the film bulk, and particularly the bottom pure DPP layer, remains intact. GIWAXS measurements on the DPP-based films (Fig. 2e, f and Supplementary Figs. 11 and 12) reveal that both pure DPP and 50% DPP/SU8 blends have a predominant edge-on orientation of the polymer chains in accord with DPP literature reports.^27^ Specifically, along the in-plane direction (Fig. 2e and Supplementary Table 1), the typical broad (010) DPP reflection at 1.65 ± 0.0011 Å^−1^, corresponding to a π–π stacking distance (*d*_*π*_) of 0.38 ± 0.0004 nm, is weakened in the 50%DPP/SU8 films. However, other small peaks (1.48/1.55/1.62/1.72 Å^−1^) present in the CHCl_3_-developed and soaked 50%DPP/SU8 films are assigned to DPP nanofibers.^28^ Thus, SU8 promotes DPP aggregation in solution or during the film deposition, yielding fibers with polymer chains π–π stacked at ~0.4 nm distances. This morphology is known to enhance conjugation length and carrier mobility in π-polymers.^29,30^ Furthermore, a (100) reflection appears in all blends at ~0.30 nm (absent in pristine DPP films), corresponding to lamellar spacings of *d*_*lam*_ = 2.07–2.13 nm. This may reflect formation of a mixed orientation morphology in blending (Supplementary Table 2). Along the out-of-plane direction (Fig. 2f), all films exhibit a family of (*n*00) reflections corresponding to a lamellar stacking located at ~0.32 Å^−1^ and corresponding to a *d*_*lam*_ of 1.93–1.99 nm. The lamellar stacking coherence lengths (*ξ*) for all films are summarized in Fig. 2g and Supplementary Tables 1–4.^31^ The *ξ* of the as-deposited DPP is 24.6 nm and falls to 16.8 nm for the uncured 50% DPP/SU8 film. The *ξ* has minimal change on UV irradiation, CHCl_3_ development, or 24 h immersion in CHCl_3_, further demonstrating the excellent UV and chemical resistance. Similar trends are observed for other x% DPP/SU8 and x% DPP/PCell blends (Supplementary Note 1, Supplementary Figs. 13–15).

AFM images of 25% DPP/SU8 films from different processing conditions in Supplementary Fig. 16, clearly reveal the presence of DPP nanofibers on the top surface, which are removed after developing in CHCl_3_. Regarding PA effects, the uncured 50% DPP/PCell films (Supplementary Fig. 17) exhibit a distinctive fibrillar surface morphology (σ_rms_ = 4.7 nm)^32,33^. The film morphology is unchanged after UV irradiation, but the film smoothens after CHCl_3_ development for 5 s (σ_rms_ = 3.1 nm) and eventually the fibrous morphology disappears after 24 h CHCl_3_ immersion, yielding a fish scale morphology. By decreasing (25% DPP/PCell) or increasing (75% DPP/PCell) the semiconductor contents in the blend, similar morphological evolutions are noted (Supplementary Fig. 18). In the case of N2200-based films (Supplementary Figs. 9 and 19), pure N2200 films exhibit a typical smooth surface with σ_rms_ of only 0.3 nm.^34^ The 50% N2200/SU8 films also exhibit a fibrillar morphology and σ_rms_ = 2.1 nm; however, the fibrous structure decreases on UV irradiation and the film exhibits a σ_rms_ = 0.8 nm. After development, these films also exhibit a fish scale morphology with a greatly increased σ_rms_ = 3.5 nm again due to partial removal of the N2200/SU8 discontinuous phase^33^. Note that selective removal of SU8 or PCell from the uncured blends with the developing solvent (See Experimental Section for details) leaves ~15 nm thick fibrillar DPP or N2200 films consistent with a semiconductor-rich bottom interface (Supplementary Fig. 20), demonstrating the VPS between semiconductors and PAs^20,25,33,35,36^. Impressively, these fibrillar films, exhibiting good phase purity and connectivity, are OTFT-active (Supplementary Fig. 21).

Regarding the GIWAXS results for N2200-based films, pure N2200 films (Supplementary Fig. 22) exhibit typical preferential π-face-on polymer crystallite orientation with a broad π-stacking peak (010) at 1.61 ± 0.0012 Å^−1^ (*d*_π_ = 3.9 ± 0.003 Å) known for this polymer.^37^ The in-plane plot shows four orders of lamellar reflection with the lowest order (100) located at 0.25 ± 0.0002 Å^−1^ (*d*_lam_ = 2.48 ± 0.004 nm) and three orders of (001) backbone periodicity the first located at 0.46 ± 0.0011 Å^−1^ (*d*_back_ = 1.38 ± 0.007 nm).^38^ When N2200 is blended with SU8 at 50%, the out-of-plane (010) and in-plane (00n) reflections are strongly suppressed vs. that of neat N2200 while the other reflections, such as the (n00) reflections, persist or even increase in intensity (Supplementary Figs. 23 and 24). However, comparing the plots proceeding from uncured to soaked blend films, the patterning process has minimal effect on the N2200 macromolecular packing and texturing. Thus, the *ξ* of the (100) peak for the 50% N2200/SU8 films (Fig. 2g, Supplementary Table 5) slightly decreases from 21.4 ± 0.6 nm (as-deposited) to 18.7 ± 0.5 nm (UV cured), then to ~17.4 nm for both the developed and 24 h immersion in SU8 developer films. Similar trends are observed for other x% N2200/SU8 and 50% N2200/PCell blends (Supplementary Figs. 22 and 25). These comprehensive characterizations for various semiconductor/PA films demonstrate that blending with the PA, as well as curing and development, does not compromise semiconductor film texturing. Furthermore, all blends remain macroscopically uniform and continuous. Finally, VPS and nanofiber formation during the film deposition process is confirmed. Thus, the combined characterizations demonstrate positive morphological and structural characteristics which are critically important for high-resolution patterning of the semiconductor film and efficient charge transport in the corresponding devices as verified in the next section.

### Device electrical properties and stability

**Fig. 3:**
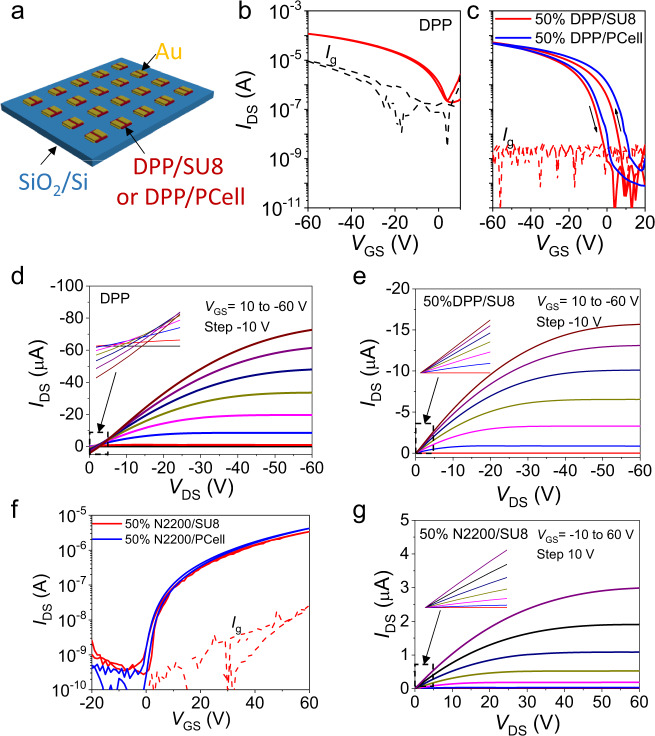
Response characteristics of patterned organic p- and n-type transistors. **a** Schematic OTFT structure using patterned DPP-based films and evaporated Au source/drain electrodes. Transfer curves of OTFTs based on **b** pristine DPP, **c** patterned 50% DPP/SU8 and 50% DPP/PCell films. Output curves of OTFTs using, **d** pristine DPP, **e** patterned 50% DPP/SU8 films. **f** Transfer curves of OTFTs based on 50% N2200/SU8 and 50% N2200/PCell films. **g** Output curves of OTFTs based on patterned 50% DPP/SU8 films. All devices here have *W*/*L* of 1000/100 μm.

Next, the performance of the DPP BGTC and N2200 TGBC OTFTs was evaluated, starting with large channel (*W/L* = 1000/100 μm) devices based on pristine/unpatterned DPP as well as developed/patterned x% DPP/SU8 and x% DPP/PCell films (Fig. 3a). Note that the DPP-based devices exhibit typical *p*-type behavior as seen in the transfer and output plots of Fig. 3b–e. The DPP OTFTs exhibit very high off-currents (in the level of 10^−7^ A), large subthreshold swings (*SS* = 5.9 ± 0.3 V dec^−1^), and small current on/off ratios (10^2^–10^3^), reflecting gate/parasitic leakage currents typical of high-mobility/unpatterned semiconductors^39,40^. In contrast, the patterned 50% DPP/SU8 and 50% DPP/PCell devices (semiconductor area = 1.1 × 1.3 mm^2^) exhibit low off/gate currents (10^−10^ A, instrumentation-limited), lower *SS* (2.2 ± 0.2 V dec^−1^), high current on/off ratios (>10^6^) and textbook output curves. Electrical parameters were extracted using standard MOSFET equations and are summarized in Table 1. The average carrier mobility (*μ*) and threshold voltage (*V*_T_) for the DPP devices are 0.4 ± 0.05 cm^2^ V^−1^ s^-1^ and 20.2 ± 2.5 V, respectively. These values are comparable to previously reported pristine DPP data in cases where the mobility was realistically estimated^20,28,41,42^. The mobilities of the patterned 50% DPP/SU8 and 50% DPP/PCell OTFTs are 0.24 ± 0.04 cm^2^ V^−1^ s^−1^ (*V*_T_ = 18.6 ± 2.3 V) and 0.20 ± 0.03 cm^2^ V^−1^ s^−1^ (*V*_T_ = 29.7 ± 2.8 V), respectively, indicating minimal degradation during patterning. Varying the DPP content in the blend to lower (25%) and higher (75%) values does not significantly change the transport characteristics or field-effect mobility, which remains in the range of 0.1–0.22 cm^2^ V^−1^ s^−1^ (Supplementary Figs. 26 and 27). Interestingly, the *V*_T_s for the 25%DPP/SU8 and 25% DPP/Cell OTFTs are much lower than those of the pristine DPP and other DPP-based blends. Seen from Supplementary Table 2, we believe that when the DPP concentration is lower than a critical point (perhaps percolation related), the edge-on packing contribution (62.6% for 25%DPP/SU8) is much lower than those of 50%DPP/SU8 (94.9%) and pristine DPP (99.6%). Thus, different switching behavior is observed at high *V*g (shown in the transfer curves, Supplementary Fig. 27), resulting in much lower *V*_T_s. Finally, DPP and 25% DPP/SU8 OTFTs with TGBC and BGBC (bottom-gate bottom-contact) architectures were also evaluated. However, they all exhibit poor performance likely due to unfavorable charge injection from pristine Au electrodes into DPP in bottom-contact devices (Supplementary Fig. 28).

**Table 1:** Performance metrics of selected TFTs based on pristine semiconductor and patterned polymer blend films^a^.

Semiconductor	Device structure	*W/L* (μm/μm)	µ (cm^2^ V^−1^ s^−1^)	*I*_on_:*I*_off_	*V*_T_ (V)	*SS* (V dec^−1^)
DPP	BGTC	1000/100	0.4 ± 0.05	<10^3^	20.2 ± 2.5	5.9 ± 0.3
25% DPP/SU8			0.1 ± 0.02	10^5^–10^6^	0.63 ± 0.1	2.4 ± 0.2
50% DPP/SU8			0.24 ± 0.04	10^5^–10^6^	18.6 ± 2.3	2.2 ± 0.2
75% DPP/SU8			0.22 ± 0.03	10^5^–10^6^	20.0 ± 2.6	2.3 ± 0.1
25% DPP/Cell			0.1 ± 0.02	10^5^–10^6^	7.1 ± 0.5	2.7 ± 0.2
50% DPP/Cell			0.2 ± 0.03	10^5^–10^6^	29.7 ± 2.8	2.4 ± 0.1
75% DPP/Cell			0.2 ± 0.02	10^4^–10^5^	25.4 ± 2.0	3.2 ± 0.2
50% DPP/SU8		40/10^b^	0.1 ± 0.02	10^5^–10^6^	11.3 ± 1.4	1.4 ± 0.08
Ultraflexible		1000/100	0.03 ± 0.01	10^3^–10^4^	20.5 ± 3.2	3.7 ± 0.4
50% DPP/SU8						
N2200	TGBC	1000/100	0.1 ± 0.02	10^4^–10^5^	9.9 ± 1.0	5.3 ± 0.3
25% N2200/SU8			0.06 ± 0.01	~10^5^	18.3 ± 2.3	3.0 ± 0.2
50% N2200/SU8			0.09 ± 0.02	10^4^–10^5^	5.6 ± 0.5	2.1 ± 0.2
50% N2200/SU8			0.08 ± 0.02	~10^4^	6.8 ± 0.5	3.0 ± 0.3
(THF)						
50% N2200/Cell			0.1 ± 0.02	10^4^–10^5^	8.3 ± 0.7	2.0 ± 0.1
Ultraflexible		1000/50	0.01 ± 0.005	10^4^–10^5^	17.9 ± 2.9	3.6 ± 0.4
50% N2200/SU8						

^a^Data collected from >15 devices. Chloroform used solvent unless otherwise indicated.

^b^W/L defined by lift-off process.

Regarding the pristine N2200, x% N2200/SU8, and x% N2200**/**PCell TGBC devices, Figs. 3f, g and Supplementary Fig. 29 show representative transfer and output curves. The patterned devices exhibit optimal *I–V* characteristics with lower off-currents (10^−10^ A), lower gate currents (10^−8^ A), lower *SS* (~2.0 V dec^−1^), and near-zero turn on voltages vs. the unpatterned N2200 TFTs, while the electron mobilities of 0.09 ± 0.02 cm^2^ V^−1^ s^−1^ are comparable to those of literature N2200 devices.^43^ To avoid the toxic chloroform solvent used above and to make this fabrication and patterning process potentially acceptable in semiconductor FABs, the green solvent THF was used as proof-of-concept for 50% N2200/SU8 films. It is found that these films exhibit satisfactory patterning and electrical performance (Supplementary Fig. 30). Note, we also attempted to fabricate DPP TFTs with THF, however, this semiconductor is not sufficiently soluble in this solvent for processing.

**Fig. 4:**
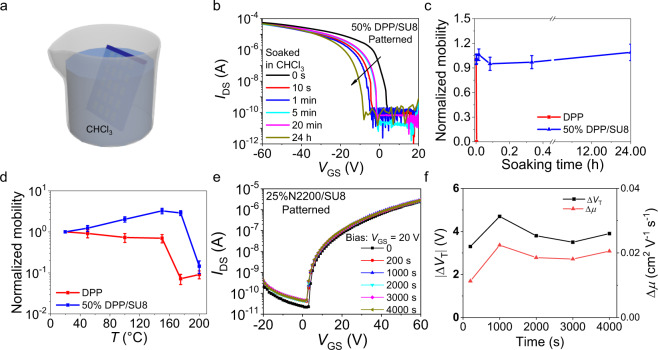
Chemical and thermal stability of DPP and 50% DPP/SU8 devices. **a** Schematic of device soaking in CHCl_3_. **b** Transfer curves and **c** normalized mobility of DPP and patterned 50% DPP/SU8 devices after CHCl_3_ immersion for various times. The *V*_DS_ is −60 V. The error bars are the standard deviation. **d** Normalized mobilities of DPP and 50% DPP/SU8 devices evaluated at various temperatures. The error bars are the standard deviation. **e** Transfer curves evolution of patterned 25%N2200/SU8 OTFTs under positive gate bias stress at a *V*_GS_ of 20 V for up to 4000 s. The *V*_DS_ is 60 V. **f** Threshold voltage shift (Δ*V*_T_) and mobility shift (Δµ) of the patterned 25%N2200/SU8 devices OTFTs under positive gate bias stress.

Next, we evaluated the chemical, thermal and bias stabilities of our patterned OTFTs vis-à-vis those based on the pristine semiconductors. As noted in the morphology and GIWAXS sections above, the DPP/SU8 films are stable upon long-term exposure to CHCl_3_. Thus, we investigated how the corresponding TFTs, fabricated on robust Si/SiO_2_ gate contact/dielectric platforms respond to a similar treatment. The data in Figs. 4a-3c indicate that while the DPP TFTs are immediately damaged upon immersion in CHCl_3_, the patterned 50% DPP/SU8 devices function even after CHCl_3_ immersion for 24 h, but with the transfer curve shifting negative with increasing immersion time and pinned maximum on-current and carrier mobility. The negative shift probably originates from additional charge traps due to solvent intercalation and nanoscopic morphological changes in the semiconductor during extended solvent immersion. Regarding the thermal stability, blending organic semiconductors with high glass-transition temperature (*T*_g_) insulating polymers has proved to be an efficient way to improve film morphological and device thermal stability.^44^ Considering the high SU8 *T*_g_ (~200 °C), we next compared the performance changes of OTFTs in ambient based on DPP and 50%DPP/SU8 films. As shown in Fig. 4d and Supplementary Fig. 31, the 50% DPP/SU8 devices retain a high µ of 0.7 ± 0.1 cm^2^ V^−1^ s^−1^ upon thermal annealing up to 175 °C, while the DPP ones do not function at temperatures higher than 150 °C.

The operational stability of representative N2200/SU8 and DPP/SU8 OTFTs was also probed by applying a gate voltage (*V*_GS_ = ±20 V) for up to 4000 s (Figs. 4e, f, Supplementary Fig. 32). The results show that TGBC 25%N2200/SU8 devices exhibit excellent bias stability with threshold voltage shift (Δ*V*_T_) and mobility shift (Δ*µ*) below 4 V and 0.02 cm^2^ V^−1^ s^−1^, respectively. BGTC patterned 50%DPP/SU8 devices are also tested and exhibit larger Δ*V*_T_ (~16 V) and Δµ (~0.06 cm^2^ V^−1^ s^−1^) variation than the DPP devices during the bias test (*V*_GS_ = −20 V for up to 4000 s). However, note that pristine BGTC DPP TFTs exhibit a similar stress behavior [Δ*V*_T_ (~12 V) and Δµ (~0.05 cm^2^ V^−1^ s^−1^)]. These data indicate that, as expected, a top-gate architecture better stabilizes devices during bias stress in ambient and, more importantly, the addition of PA does not impact the device stability of either device.

### High-resolution patterning and ultraflexible devices

**Fig. 5:**
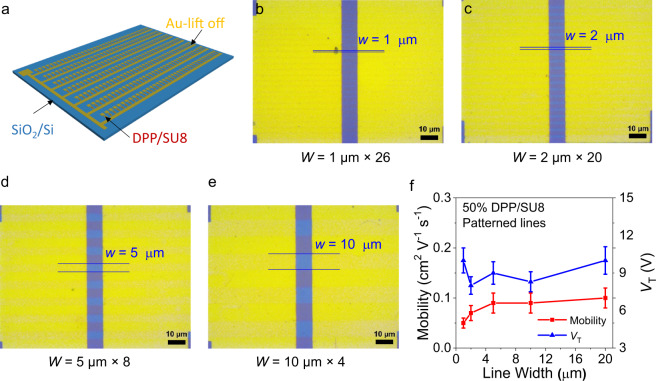
High-resolution patterned DPP/SU8 lines and corresponding device performance. **a** Schematic of TFT arrays based on patterned DPP/SU8 films and photolithographically defined (lift off) Au source/drain electrodes. **b**–**e** Optical images of patterned 50% DPP/SU8 lines (horizontal) and Au contacts (vertical) on SiO_2_/Si substrates. The scale bars are 10 μm. **f** Mobility and threshold voltage evolution of 50% DPP/SU8 devices on OTS-treated SiO_2_/Si substrates with varied line widths.

Owing to the excellent chemical stability of the above semiconducting polymer blends, additional BGTC device architectures were fabricated by combining patterned 50% DPP/SU8 semiconducting lines of different widths (*w* = 1–20 µm) with photolithographically patterned Au Source/Drain electrodes (layout in Fig. 5a). Note, 50% DPP/SU8 lines were fabricated as discussed above by photo exposure/CHCl_3_ development, while Au patterning used a lift-off process involving the S1813 photoresist, the aggressive AZ^®^ 400 K developer (alkaline solution), and acetone. Fig. 5b–e demonstrate that metal electrodes with channel length/width of 10/100 µm can be precisely patterned on the semiconducting lines. Representative TFT transfer plots and transport parameters of TFTs based on these channel topologies are shown in Fig. 5f and Supplementary Fig. 33. Note here that the effective channel width, *W*, of these devices is *n* × *w*, where *n* are the number of semiconducting lines in the channel area. The hole mobility of these TFTs remains 0.09 ± 0.02 cm^2^ V^−1^ s^−1^ when the line width is greater than 5 µm, then gradually falls to 0.05 ± 0.01 cm^2^ V^−1^ s^−1^ when the line width is 1 µm, likely due to the large effect of the line sidewalls. The *V*_T_ and *SS* are relatively stable at 9 ± 0.8 V and 1.4 ± 0.1 V dec^−1^, respectively. TFT arrays (100 dpi) were also fabricated with different channel lengths which show uniform device performance (Supplementary Fig. 34). These data demonstrate the realization of very robust semiconductor structures with large surface/volume ratios which should also be suitable for fabricating sensors/TFTs and electrochemical transistors.

**Fig. 6:**
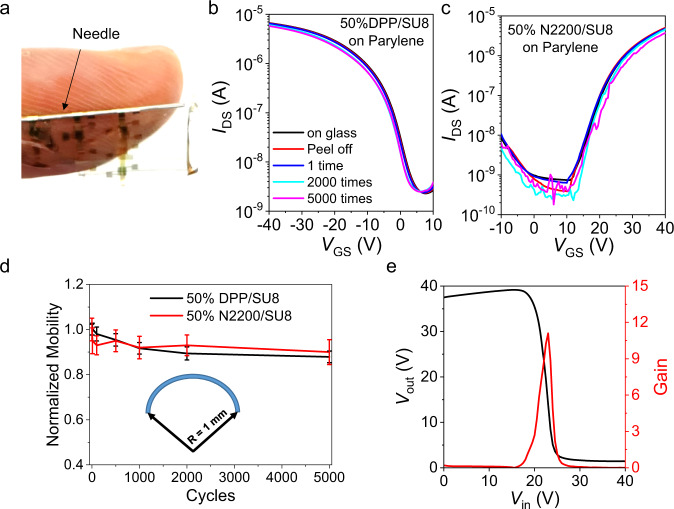
Patterned ultraflexible organic p- and n-type transistors. **a** Photo of an ultraflexible DPP/SU8 device array on a 1.5 μm thick parylene substrate. The semiconductor layers are patterned by photolithography while the S/D electrodes are patterned with metal mask. Transfer curves of ultraflexible. **b** BGTC 50% DPP/SU8 devices and **c** BCTG 50% N2200/SU8 devices before and after bending at a radius of 1 mm for the indicated repetitions. The *V*_DS_ is 40 V. **d** Mobility variations of ultraflexible 50% DPP/SU8 and 50% N2200/SU8 devices after bending at a 1 mm radius for the indicated repetitions. **e** Static switching characteristics and gain of an inverter based on ultraflexible 50% DPP/SU8 and 50% N2200/SU8 devices. The supply voltage is set at 40 V.

Finally, to further validate materials and processing generality, ultraflexible BGTC and TGBC TFT arrays based on patterned 50% DPP/SU8 and 50% N2200/SU8 films, respectively, were fabricated on 1.5 µm-thick parylene substrates. For simplicity of integration, a 300 nm-thick parylene film was also used as the gate dielectric (see parylene dielectric properties in Supplementary Fig. 35). Optical images, device structures, and representative transfer plots of these devices are shown in Fig. 6a–c and Supplementary Fig. 36. The ultraflexible 50% DPP/SU8 and 50% N2200/SU8 devices exhibit an average mobility of 0.03 ± 0.01 and 0.01 ± 0.005 cm^2^ V^−1^ s^−1^, respectively. Importantly, negligible mobility/*V*_T_ changes are observed after peeling the devices from the rigid support and bending them 5000 times at a radius of 1 mm despite microcracks forming in areas surrounding the device (Fig. 6d and Supplementary Fig. 37). Finally, Fig. 6e shows the static switching characteristics and the gain of an ultraflexible inverter based on the p- + n-TFTs. The wide-range switching voltage with a 40 V supply voltage is 22.2 ± 0.3 V, where the high noise margin and low noise margin are 13.2 ± 0.2 V and 15.6 ± 0.2 V, respectively. This inverter exhibits a gain of 11, rivalling or exceeding the performance of other solution-processed flexible devices.^45–48^

## Discussion

We demonstrate a versatile strategy for foundry-compatible high-resolution patterning of organic semiconducting films by crosslinking a vertically phase-separated blend of the semiconducting polymer and a UV photocurable additive (PA). This process is effective for both p- and n-type semiconducting polymers, can use environmentally benign solvents (e.g., THF) under ambient atmosphere, and yields ultraflexible transistor circuitry. GIWAXS, AFM, and TOF-SIMS analyses reveal formation of textured semiconductor film morphologies with a vertically multiphase-separated channel layer preserved during the patterning process. Both PAs promote the formation of close-packed DPP or N2200 nanofiber structures at the bottom of the blend film, which is crucial to achieving functional devices even when only 25 wt% of the semiconductor is added. Equally important, PA addition influences semiconductor polymer backbone orientation, especially for blends with a >50wt % PA content. Excessive PA significantly reduces the edge-on content, thus degrading device performance. Compared with TFTs based on the pristine (unpatterned) polymer semiconductors, the present polymer blend-based patterned devices exhibit optimal transfer/output curves, higher thermal/chemical stability, respectable p-type and n-type TFT performance, and enable the fabrication of ultraflexible TFTs and complementary inverters. Note that the TFT mobilities reported here are not limited by the fabrication methodology, which should be readily applicable to current generation semiconducting polymers.^49–52^ A possible limitation of the present patterning method relying on VPS is the limited charge transport along the vertical direction since the PA-rich layer is required to protect the underlying semiconducting polymer layer during photolithography. Thus the addressable patterning of high-performance vertical devices, such as organic photovoltaic devices, photodetectors and light-emitting diodes, may be more challenging. Solution-processed organic electronics, especially those enabled by printing technologies, are on the verge of large-scale industrialization. Thus, we can envision that the continuous development of high-performance semiconductors compatible with environmentally benign solvents, together with the present patterning methodology, will facilitate the industrialization of solution-processed organic electronics.^53,54^

## Methods

### Materials

Poly[2,5-bis(2-octyldodecyl)–3,6-diketopyrrolopyrrole-alt-5,5’-(2,5-di(thien-2-yl)thieno[3,2-*b*]thiophene)] (abbreviated as DPP) was purchased from 1-Material Inc.. Poly[[*N, N*-bis (2-octyldodecyl)-napthalene-1, 4, 5, 8-bis (dicarboximide)–2, 6-diyl]-alt–5,5’-(2,2’-bithiophene)](P(NDI2OD-T2), N2200) was provided by Flexterra Inc.. Trichloro(octyl)silane (OTS), polymethyl methacrylate (PMMA, average *M*_w_ ~120 kg/mol), 2-butanol, acetone, Tetrahydrofuran and anhydrous chloroform were purchased from Sigma-Aldrich. Microposit^TM^ S1813^TM^ photoresist, SU-8 2000.5 photoresist, SU-8 2002 photoresist, and SU8 developer were purchased from Kayaku Advanced Materials, Inc. (formerly MicroChem Corp.). AZ^®^ 400 K developer and hexamethyldisilazane (HDMS) adhesion promoter were purchased from Microchemicals GmbH. The 300 nm-thick SiO_2_/Si substrates and Corning^®^ glass substrates were purchased from University Wafer Inc. and Ted Pella Inc., respectively.

### Synthesis of PCell

PCell was synthesized according to our previously published procedure.^19^ Specifically, a suspension of 1.0 g of cellulose in 40 mL of N, N-dimethylacetamide was kept at 130 °C for 2 h under stirring. After the slurry was allowed to cool to 100 °C, 3.0 g of anhydrous lithium chloride was added. The cellulose was completely dissolved as the solution was cooled to room temperature under stirring. Next, the cellulose solution was put into an ice bath for 15 min, followed by adding 3.2 g of cinnamoyl chloride. The reaction mixture was heated at 80 °C for 24 h before pouring into an excess volume of ethanol (100 mL). The precipitate was collected by filtration and then it was extracted with ethanol in a Soxhlet extractor for 12 h. Finally, we obtained the dried PCell with a yield of 84.3% after drying under vacuum at 50 °C.

### Preparation of solutions and substrates

Exactly 8 mg of DPP, 8 mg of N2200, and 8 mg of cinnamate-functionalized PCell were separately dissolved in 2 mL of chloroform. Next, 50 µL SU-8 2000.5 photoresist was diluted with 1.89 mL of chloroform to form a 4 mg/mL solution. All the solutions were stirred overnight at 50 °C in a glovebox. PMMA/2-butanol solutions were prepared by dissolving 70 mg of PMMA in 1.0 mL of 2-butanol and stirring it at 60 °C for 3 h. With regard to the preparation of OTS-treated SiO_2_/Si substrates, 200 mL of OTS was dissolved in a 100 mL mixed solvent of chloroform and hexane with a volume ratio of 3:7. The clean SiO_2_/Si substrates were first treated with an O_2_ plasma for 5 min and then immersed in the above OTS solution for 3 h. Clean Corning^®^ glass substrates were used for top-gated N2200-based devices without additional cleaning.

### Fabrication of DPP and DPP/SU8 films

Approximately 1 h before device fabrication, the DPP/chloroform and SU8/chloroform solutions were mixed in a volume ratio of 1:3, 1:1, and 3:1, for fabricating the 25%, 50%, and 75% DPP/SU8 blends, respectively. The mixed solution was spin-coated on OTS-treated SiO_2_/Si substrates at 1500 rpm for 30 s in ambient (RH~30%). After prebaking at 95 °C for 1 min, the films were exposed to 365 nm UV light (F300S, Inpro Technologies, dosage = 60–80 mJ cm^−2^) through a photomask, followed by annealing at 95 °C for 2 min. For high-resolution patterning, maskless aligner (Heidelberg MLA150) equipped with 375 nm laser light was used. Next, the resulting cured films were developed in chloroform for 5 s to achieve the desired patterns. After that, the films were annealed at 150 °C for 30 min in a glovebox. The final film thickness is 40–70 nm depending on the DPP/SU8 ratio. DPP films were prepared by spin-coating of DPP/CHCl_3_ solutions at 1500 rpm for 30 s in ambient (RH~30%), followed by thermal annealing at 150 °C for 30 min in a glovebox. The film thickness of DPP film is ~120 nm. Note here the use of low boiling point (<120 °C) solvent is critical for complete VPS, and protecting the underlying polymer semiconductors during photolithography. Other high boiling point solvents such as chlorobenzene, dichlorobenzene, which were generally used in previous reports on semiconductor/insulating polymer blends, are unsuccessful for efficient patterning.^20–26^

### Fabrication of DPP/PCell films

About 1 h before device fabrication, DPP/chloroform solution and PCell/chloroform solution were mixed with a volume ratio of 1:3, 1:1, and 3:1 for fabricating the 25%, 50%, and 75% DPP/PCell blends, respectively. The mixed solution was spin-coated on OTS-treated SiO_2_/Si substrates at 1500 rpm for 30 s in ambient (RH~30%). After prebaking at 120 °C for 1 min, the films were exposed to 365 nm UV light (dose = 60–80 mJ cm^−2^) through a photomask, followed by annealing at 120 °C for 5 min. For high-resolution patterning, maskless aligner (Heidelberg MLA150) equipped with 375 nm laser light was used. Next, the resulting cured films were developed in chloroform for 5 s to achieve desired patterns. After that, the films were annealed at 150 °C for 30 min in a glovebox. The final film thickness is 40–70 nm depending on the DPP/PCell ratio.

### Deposition and patterning of gold electrodes

For OTFTs with large channel size (*W*/*L* = 1000/100 µm), gold electrode patterns (30 nm thick) were achieved by thermal evaporation underneath semiconductor blend films through a metal mask. For OTFTs with small channel sizes (*W* = 20–200 µm *L* = 1–20 µm), a MCC Primer 80/20 layer (from MicroChem) was first spin-coated on semiconductor layer, followed by 100 °C/annealing for 60 s. S1813 photoresist was then spin-coated at 4000 rpm for 60 s and thermal annealed at 115 °C for 60 s. After that, the resulting films were exposed to 365 nm UV light (dose = 150 mJ cm^−2^) through a photomask and developed in AZ^®^ 400 K/H_2_O (v/v = 1/4) developer. For high-resolution patterning, maskless aligner (Heidelberg MLA150) equipped with 375 nm laser light was used. Finally, 30 nm-thick gold films were thermally evaporated on the films and the source/drain patterns were achieved by stripping in acetone. For DPP-based devices with TGBC structures, PMMA is used as dielectric layer (see below for details).

### Fabrication of TGBC N2200 and N2200/SU8 devices

About 1 h before device fabrication, N2200/chloroform solutions and SU8/chloroform solutions were mixed in volume ratios of 1:3, 1:1, and 3:1 for fabricating the 25%, 50%, and 75% N2200/SU8 blends, respectively. Cr/Au source/drain electrodes (2/23 nm thick) on glass substrates were first thermally evaporated through a metal mask. The mixed solution was spin-coated on the above substrates at 1500 rpm for 30 s in ambient (RH ~ 30%). After prebaking at 95 °C for 1 min, the films were exposed to 365 nm UV light (dose = 60–80 mJ cm^−2^) through a photomask, followed by annealing at 95 °C for 2 min. For high-resolution patterning, maskless aligner (Heidelberg MLA150) equipped with 375 nm laser light was used. Next, the resulting cured films were developed with SU8 developer for 5 s to achieve the desired patterns. After that, the films were annealed at 150 °C for 30 min in a glovebox. The final film thickness is 40–50 nm depending on the N2200/SU8 ratio. Pure N2200 films were also spin-coated at 1500 rpm for 30 s in ambient (RH~30%), followed by thermal annealing at 150 °C for 30 min in a glovebox. The film thickness of N2200 film is ~25 nm. Regarding the gate dielectric layer, a PMMA/2-butanol solution was spin-coated on the N2200 or N2200/SU8 films at 1500 rpm for 60 s in a glovebox, followed by 80 °C/3 h annealing. Finally, the gate electrodes were thermally evaporated through a metal mask to obtain bottom-contact top-gate OTFTs. Regarding the use of the green solvent tetrahydrofuran (THF), the fabrication method is identical to above procedure except that the solvent is replaced with THF and SU8 developer is diluted with IPA (SU8 developer: IPA = 7:3 in vol).

### Fabrication of TGBC N2200/PCell devices

About 1 h before device fabrication, N2200/chloroform solution and PCell/chloroform solution were mixed in volume ratios of 1:3, 1:1, and 3:1 for fabricating the 25%, 50%, and 75% N2200/PCell blends, respectively. Cr/Au source/drain electrodes (2/23 nm thick) on glass substrates were first thermally evaporated through a metal mask. The mixed solution was spin-coated on above substrates at 1500 rpm for 30 s in ambient (RH~30%). After prebaking at 120 °C for 1 min, the films were exposed to 365 nm UV light (dose = 60–80 mJ cm^−2^) through a photomask, followed by annealing at 120 °C for 5 min. For high-resolution patterning, maskless aligner (Heidelberg MLA150) equipped with 375 nm laser light was used. Next, the resulting cured films were developed in SU8 developer for 5 s to achieve the desired patterns. After that, the films were annealed at 150 °C for 30 min in a glovebox. The final film thickness is 40–50 nm depending on the N2200/SU8 ratio. PMMA dielectric and top gate electrodes were deposited by following the above procedure.

### Fabrication of ultraflexible BGTC DPP/SU8 BGTC devices

A fluorinated polymer solution [Novec 1700 and 7100 (v/v = 1:7), 3 M] was spin-coated on solvent-cleaned glass substrates, serving as a delamination layer, and next a 2 µm-thick parylene film was deposited with an SCS Labcoter^®^ 2 (PDS2010) deposition system. Next, 50 nm-thick bottom-gate electrodes were thermally evaporated though a metal mask, followed by deposition of a 300 nm-thick parylene film as the gate dielectric layer. After that, patterned 50% DPP/SU8 films and S/D electrodes (*W*/*L* = 1000/100 µm) were deposited following the procedure used for the rigid devices. Finally, the entire device arrays were delaminated from the glass/fluorinated polymer carrier prior to electrical measurements and bendability tests.

### Fabrication of ultraflexible TGBC N2200/SU8 TGBC devices

A fluorinated polymer solution [Novec 1700 and 7100 (v/v = 1:7), 3 M Company] was spin-coated on solvent-cleaned glass substrates, serving as a delamination layer, next a 1.5 µm-thick parylene film was deposited with an SCS Labcoter^®^ 2 (PDS2010) deposition system. Cr/Au source/drain electrodes (2/23 nm-thick) on glass substrates were thermally evaporated and defined by photolithography as described above. The W/L is 1000/50 µm. After that, patterned 50% N2200/SU8 films were deposited following the procedure used for the rigid devices, followed by evaporation of a 300-nm-thick parylene film as a dielectric layer. The devices were finished by depositing 50-nm-thick top-gate Au electrodes. Finally, the entire device arrays were delaminated from the glass/fluorinated polymer carrier prior to electrical measurements and bendability tests.

### Film and device characterization

Film morphologies were measured with a Bruker Dimensional Icon AFM system in the tapping mode. To acquire the bottom semiconductor morphologies, the spin-coated films (DPP/SU8, DPP/PCell, N2200/SU8 and N2200/PCell) were immersed in SU8 developer for 5 s and then rinsed with IPA; thus SU8 or PCell can be selectively removed. Optical images were taken using a Nikon Eclipse E200 microscope and film thickness is measured by a Dektak 150 surface profilometer (Veeco Instruments, Inc.). GIWAXS measurements were performed at Beamline 8ID-E at the Advanced Photon Source (APS) at Argonne National Laboratory. Samples were irradiated with a 10.9 keV X-ray at an incidence angle 0.13°–0.15° in vacuum for two summed exposures of 2.5 s (totaling 5 s of exposure), and scattering X-rays were recorded by a Pilatus 1 M detector located 228.16 mm from the sample. The collected images were then processed by using the GIXSGUI software. The background was subtracted by fitting the curves to an exponential decay, and peaks were fitted to an intermediate Lorentzian. The peak width and positions were used to calculate the correlation length and layer spacing. The coherence length was calculated using a modified Scherrer analysis which accounts for instrument resolution using the standard shape factor (*K*) = 0.866 for lamellar polymer aggregates. For DPP-based films, the second order reflection (~0.62 Å^−1^) in the out-of-plane plot is chosen for calculation of coherence length as the first-reflection is obviously affected by beam signal and the 3rd/4th order reflections are relatively weak. The electrical measurements on the dielectrics, OTFTs, and inverters were performed under ambient condition using an Agilent B1500A semiconductor parameter analyzer. The carrier mobility (*μ*) was evaluated in the saturation region. The areal capacitance for 300 nm SiO_2_/Si is 10.5 nF cm^−2^ here, while the areal capacitances of the parylene dielectric is calculated to be 8.6 nF cm^−2^. To test the chemical stability of 50% DPP/SU8 films, OTFTs based on DPP and patterned 50% DPP/SU8 films on 300 nm SiO_2_/Si substrates were immersed in CHCl_3_ for 10 s, then the devices were annealed at 100 °C for 1 min in ambient before testing to remove the remaining solvent inside the films. After that, the procedure of immersing, annealing and testing was repeated for several times until the total immersing time reached 24 h. To test the thermal stability, the DPP and 50% DPP/SU8 devices were put on a hotplate. The devices were tested when the temperature increased to the desired set point.

## Supplementary information

Supplementary Information

## Data Availability

The authors declare that the all the data supporting the finding of this study are available from the corresponding authors on reasonable request.
