# Supplementary material for: Foundry-compatible high-resolution patterning of vertically phase-separated semiconducting films for ultraflexible organic electronics
Source: Nat Commun. 2021 Aug 16;12:4937. doi: 10.1038/s41467-021-25059-8 (PMC8367968; doi:10.1038/s41467-021-25059-8)
Supplement: Supplementary file 1 — Supplementary Information [file 41467_2021_25059_MOESM1_ESM.pdf]

Supplementary Information

Foundry-Compatible High-Resolution Patterning of Vertically Phase-Separated Semiconducting Films for Ultraflexible Organic Electronics

Binghao Wang,[1,2,3] Wei Huang,[2] Sunghoon Lee,[3] Lizhen Huang,[2,4] Zhi Wang,[2,5] Yao Chen,[2] Zhihua Chen,[6] Liang-Wen Feng,[2,7] Gang Wang,[2,8] Tomoyuki, Yokota,[3] Takao Someya,[3]* Tobin J. Marks,[2]* Antonio Facchetti[2,6]*

[1]Joint International Research Laboratory of Information Display and Visualization, Key Laboratory of MEMS of Ministry of Education, School of Electronic Science and Engineering, Southeast University, 2 Sipailou, Nanjing, Jiangsu 210096, China

[2]Department of Chemistry and the Materials Research Center, Northwestern University, 2145 Sheridan Road, Evanston, IL 60208, USA

[3]Department of Electrical Engineering and Information Systems, School of Engineering, The University of Tokyo, 7-3-1 Hongo, Bunkyo-ku, Tokyo 113-8656, Japan

[4]Institute of Functional Nano & Soft Materials (FUNSOM), Jiangsu Key Laboratory for Carbon-Based Functional Materials and Devices, Soochow University, 199 Ren'ai Road, Suzhou 215123, China

[5]Research Center for Engineering Technology of Polymeric Composites of Shanxi Province, School of Materials Science and Engineering, North University of China, Taiyuan, 030051, P. R. China

[6]Flexterra Inc. 8025 Lamon Avenue, Skokie, IL 60077, USA

[7]College of Chemistry, Sichuan University, 29 Wangjiang Road, Chengdu, Sichuan 610064, China

[8]State Key Laboratory for Modification of Chemical Fibers and Polymer Materials, International Joint Laboratory for Advanced Fiber and Low-Dimension Materials, College of Materials Science and Engineering, Donghua University, Shanghai 201620, China

*E-mail: someya@ee.t.u-tokyo.ac.jp; t-marks@northwestern.edu; a-facchetti@northwestern.edu.

Supplementary Note 1

Regarding the 50% DPP/PCell films (Supplementary Fig. 20), the peak positions of the out-of-plane (*n*00) reflections are similar to those of the 50% DPP/SU8 films, but the in-plane reflections corresponding to the DPP fiber structures are much stronger, consistent with the corresponding AFM images (Supplementary Fig. 11). The blends with 25% DPP/SU8 and 25% DPP/PCell exhibit some of these reflections but with lower intensity. The ξ of the uncured 50% DPP/PCell film is 19.6 ± 0.4 nm and falls after each of the photolithography steps. However, it still remains above 13.1 ± 0.5 nm after development, which is sufficient to support efficient TFT charge transport. The 25% DPP/SU8 has a ξ of 14.1 ± 0.6 nm, which undergoes minimal change after UV irradiation, $CHCl_3$ development, or 24 h immersion in $CHCl_3$, while the 25% DPP/PCell exhibits a trend similar to that of the 50% DPP/PCell films during the photolithographic process (Supplementary Figs. 21-22).

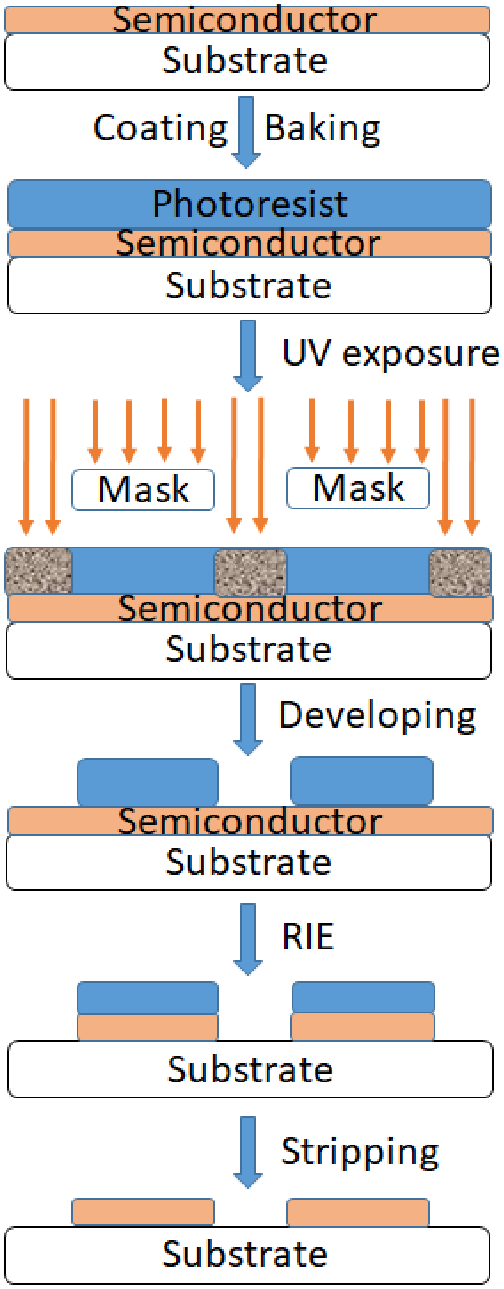

Supplementary Fig. 1. Schematic of the conventional photolithography steps for organic TFT active layers.

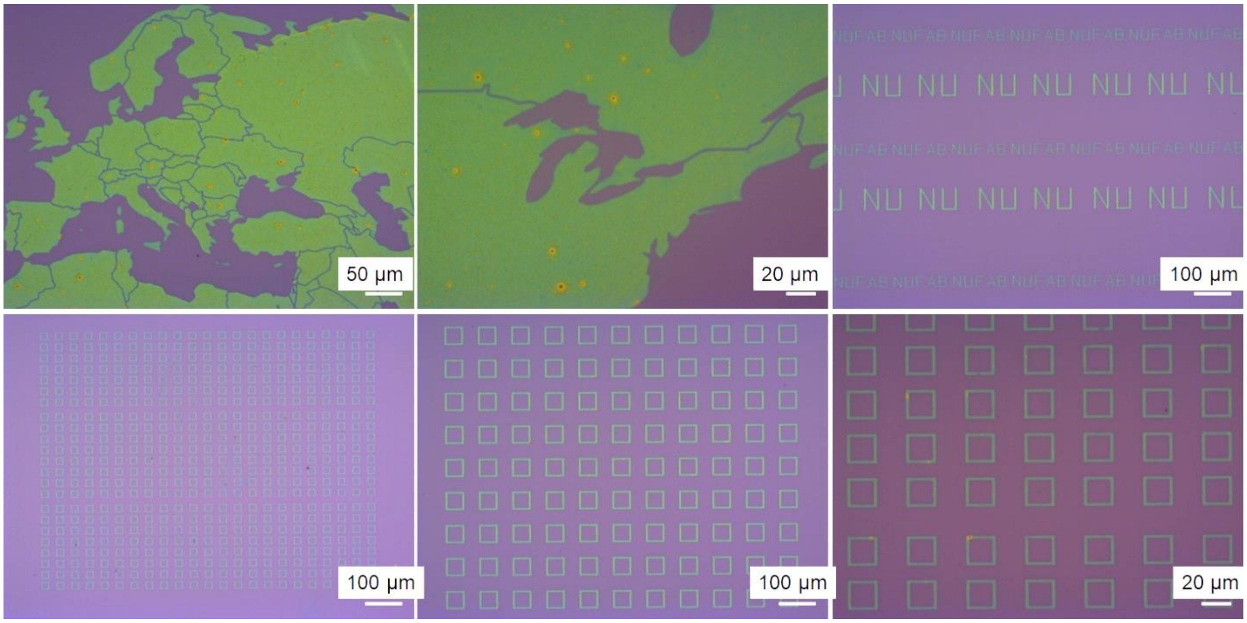

Supplementary Fig. 2. Optical images of patterned 50wt% DPP/SU8 films having different topologies.

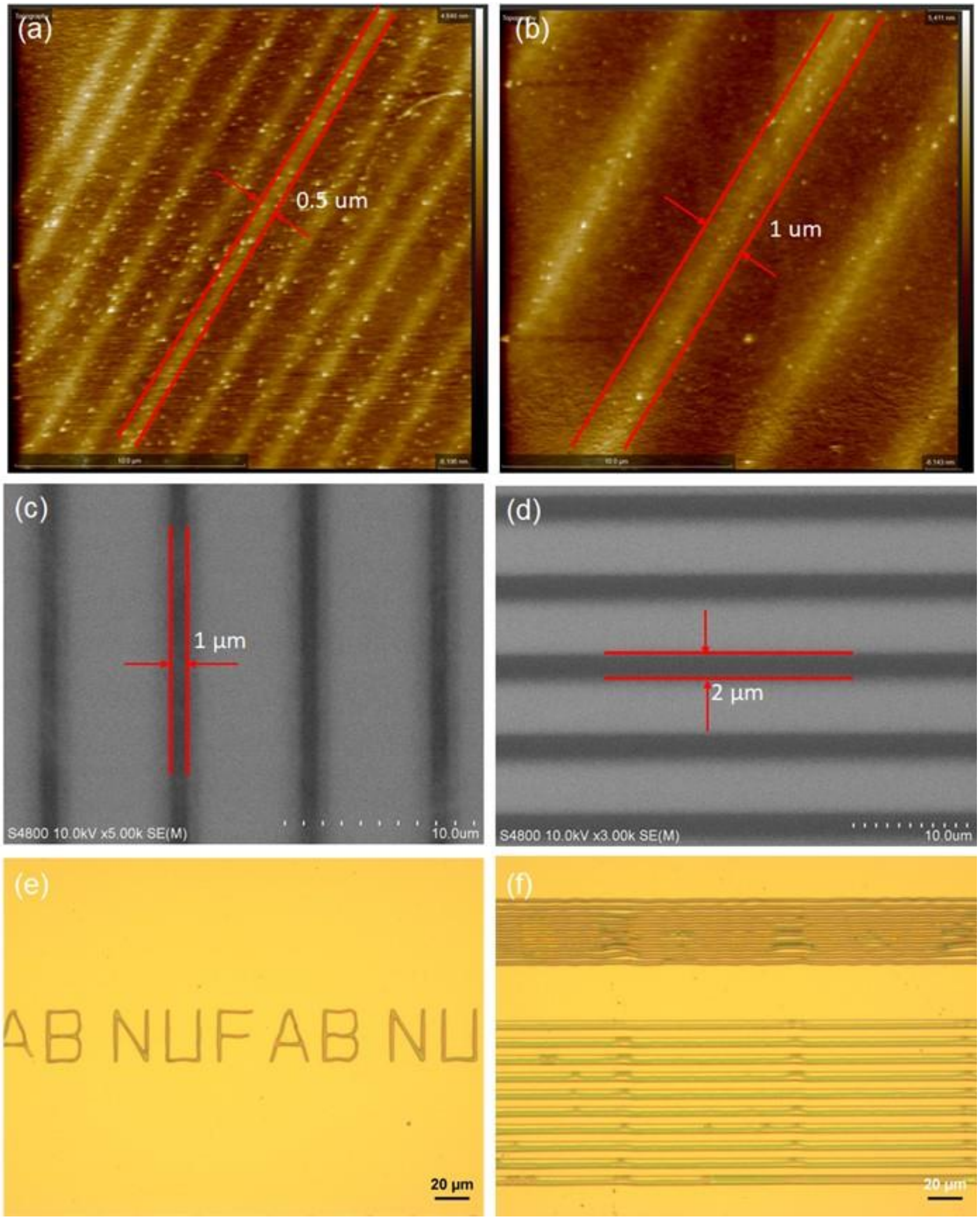

Supplementary Fig. 3. (a-b) AFM images of 50 nm-thick patterned DPP/SU8 lines. The line widths are 0.5 ± 0.01 µm and 1.0 ± 0.01 µm, respectively. (c-d) SEM images of 50 nm-thick patterned DPP/SU8 lines. (e-f) Optical images of patterned lines (~2 µm thickness) fabricated from as-received SU8 2000.5 solutions. The smallest lines have wavy shapes with widths of 2 ± 0.02 µm.

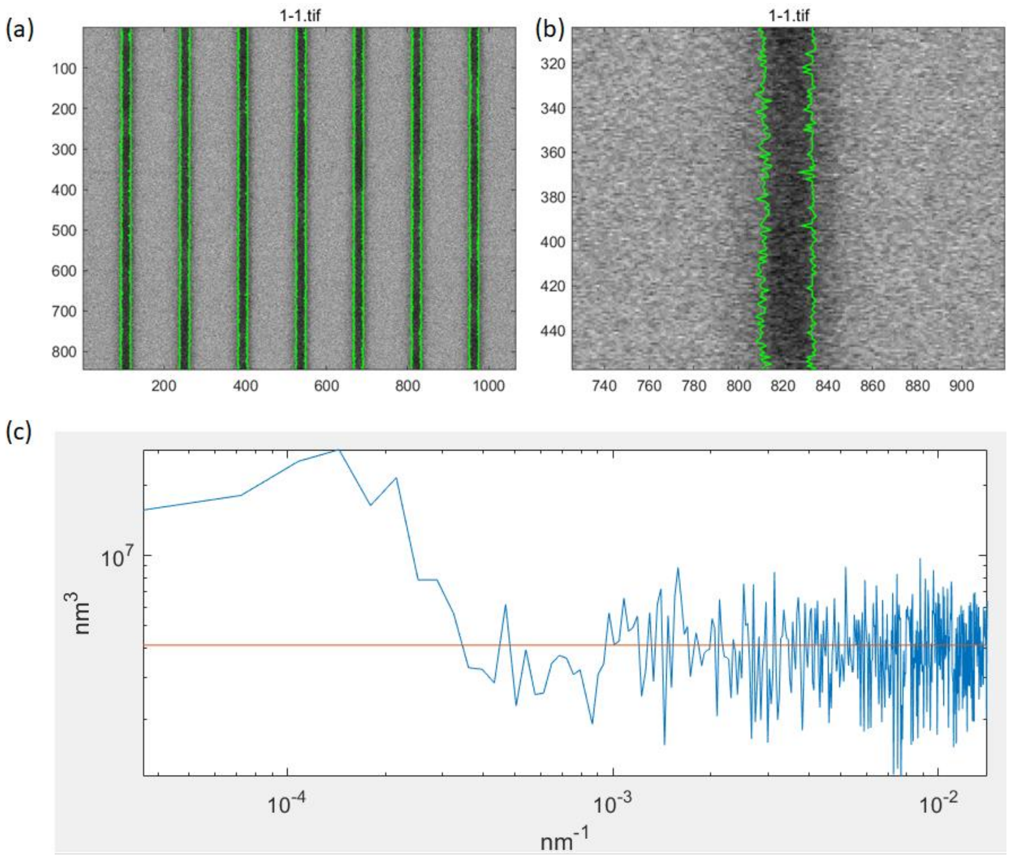

Supplementary Fig. 4 (a-b) Line width roughness (LWR) and line edge roughness (LER) simulation based on the SEM images of DPP/SU8 lines. (c) simulated power spectral density (PSD) profile. The open-access SIMLE software is used for the calculations. The mean critical dimension (CD) is 798 ± 6.5 nm with LWR and LER are 97.8 ± 1.5 nm and 70.1 ± 1.2 nm, respectively. From the PSD profile, The correlation length is 796 ± 6.2 nm and roughness exponent (α) is -1.07± 0.05 × 10^{11}. Three important equations are used for the simulation and calculation.[1, 2, 3]

$$\mathrm{LWR} = 3\sigma = 3\sqrt{\frac{1}{N}\sum_{i=1}^{N}(CD_i - \overline{CD})^2} \tag{1}$$

$$\mathrm{LWR} = \sqrt{2}\mathrm{LER} \tag{2}$$

$$\mathrm{PSD}\ (\nu) = \frac{\xi\sigma^2}{(1+\alpha\xi^2\nu^2)^{1+\alpha}} + Nl \tag{3}$$

where ξ is the correlation length, α is the roughness exponent, and Nl is the high-frequency noise background.

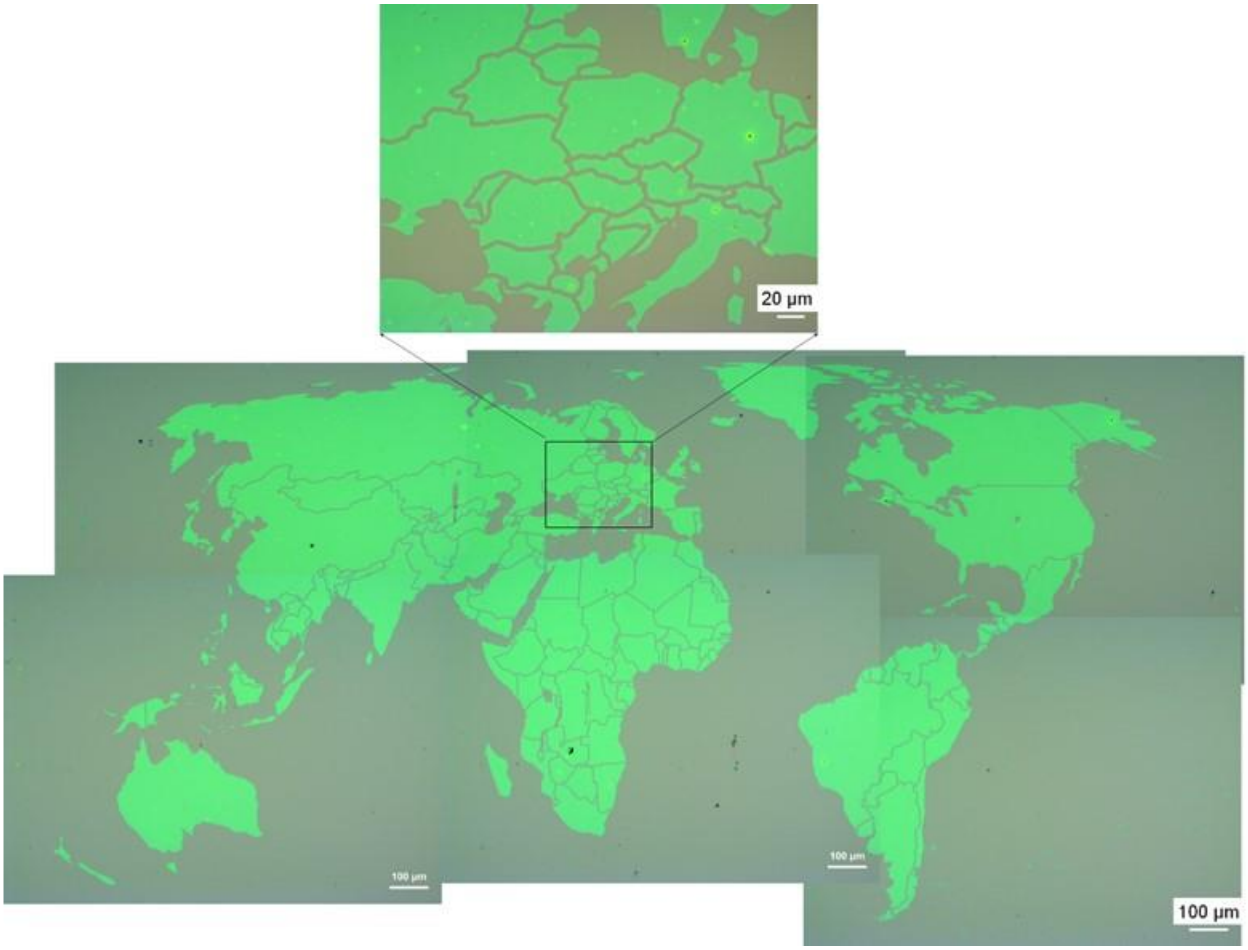

Supplementary Fig. 5 Integrated optical images of 50wt% DPP/PCell patterns, showing a global map.

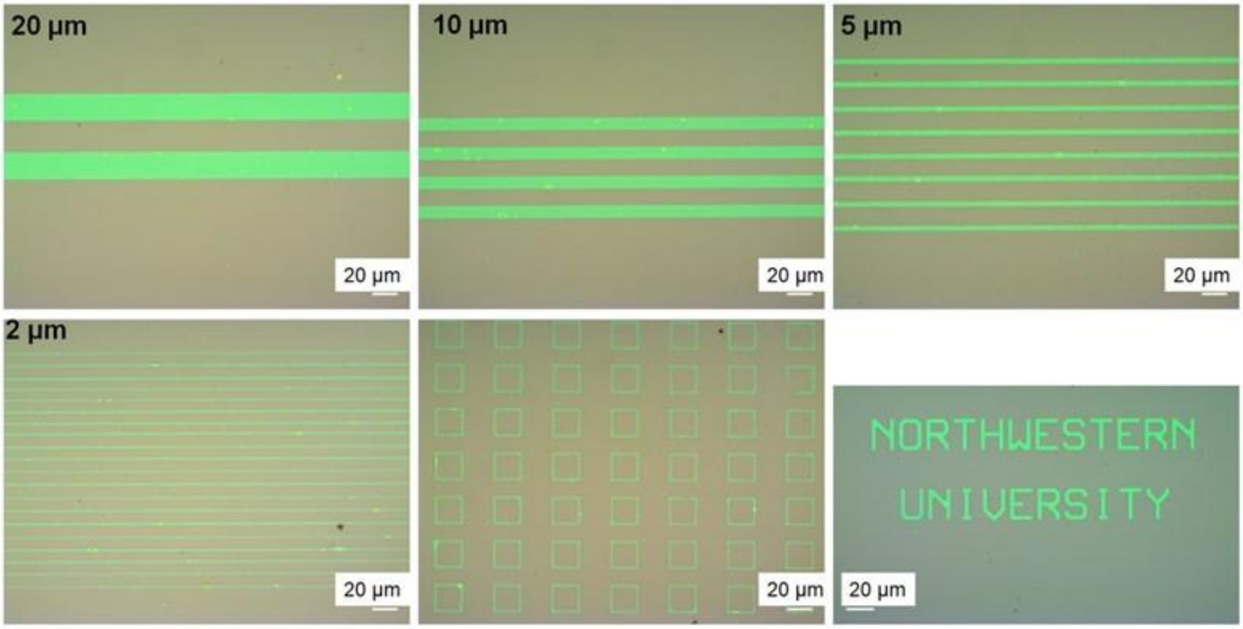

Supplementary Fig. 6 Optical images of 50wt% DPP/PCell patterns with various topologies.

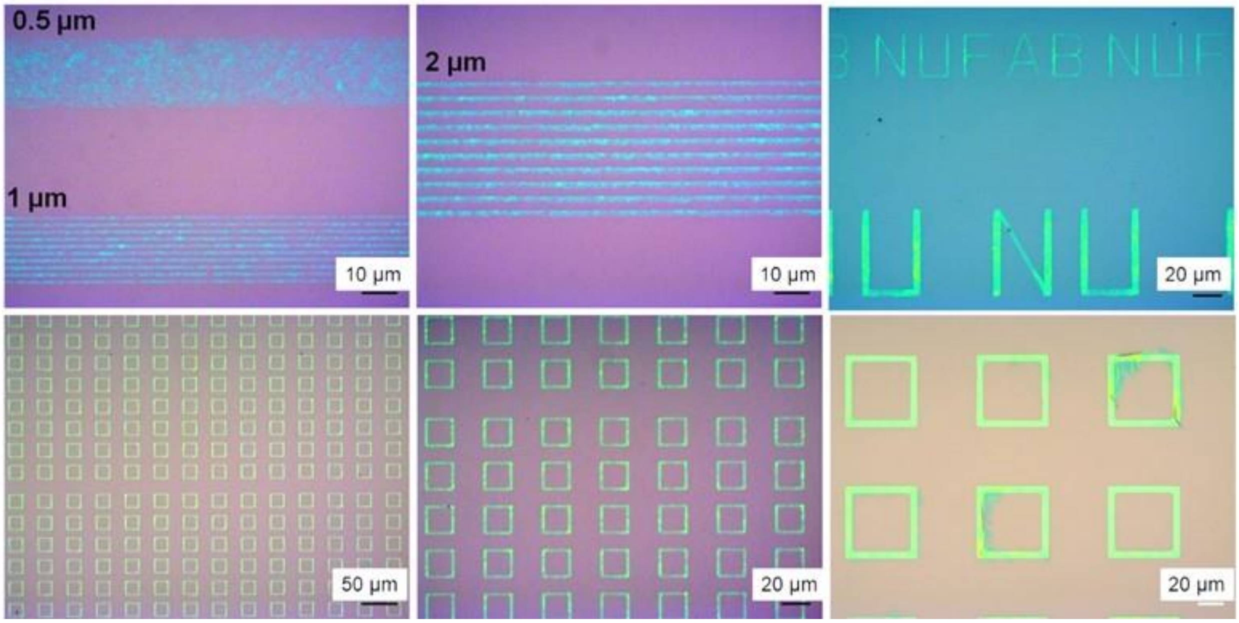

Supplementary Fig. 7 Optical images of 50wt% N2200/SU8 patterns with various topologies.

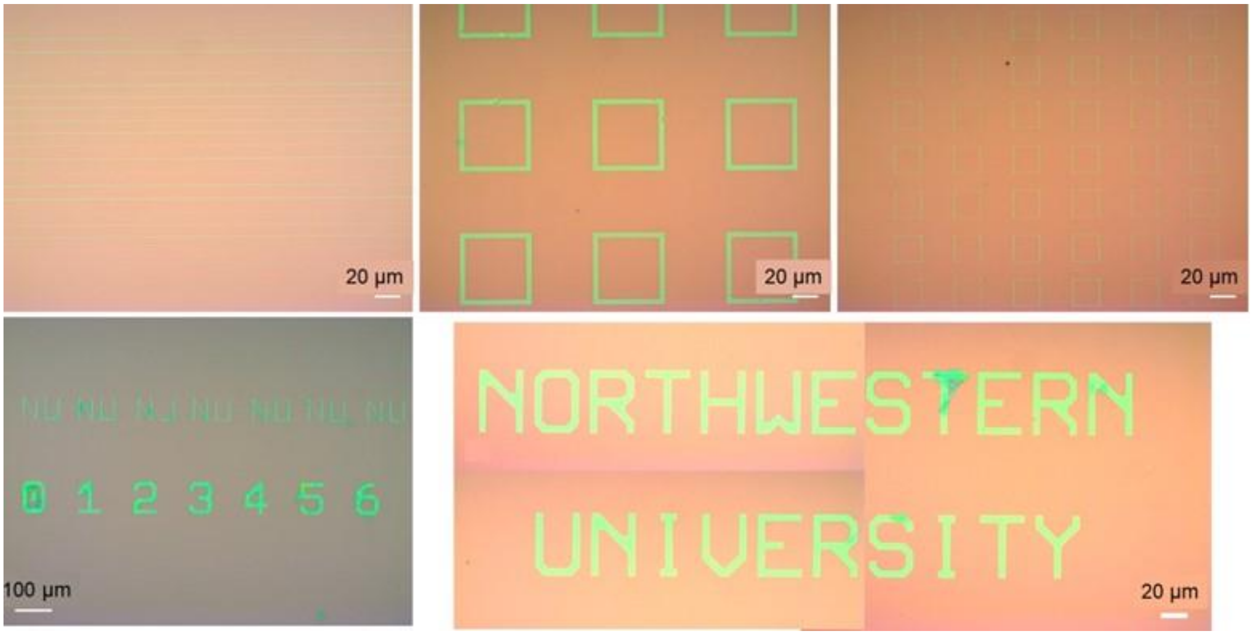

Supplementary Fig. 8. Integrated optical images of 50wt% N2200/PCell patterns with various topologies.

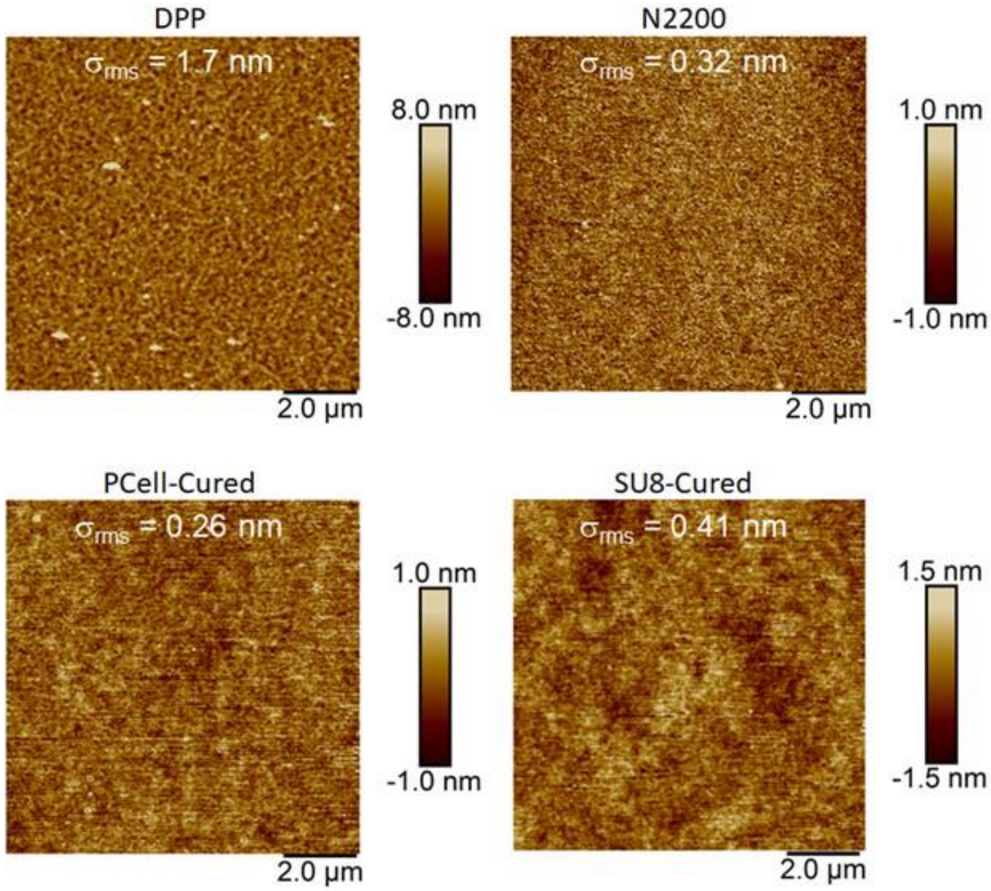

Supplementary Fig. 9. AFM images of DPP films on OTS-treated SiO_2/Si substrates. AFM images of N2200, cured PCell, and an SU8 film on SiO_2/Si substrates. The films were annealed at 150 °C for 30 min before these images were recorded.

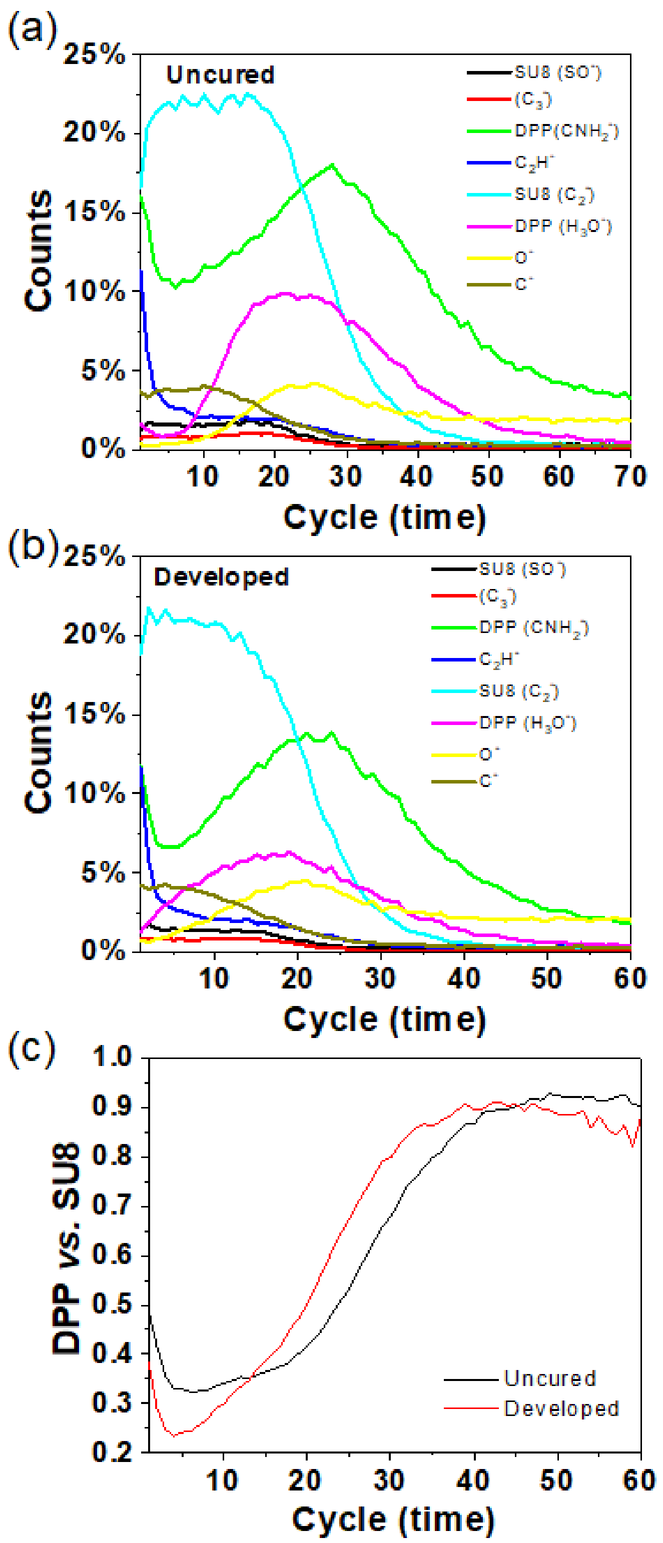

Supplementary Fig. 10. ToF-SIMS depth profile for (a) uncured and (b) developed 50% DPP/SU8 films. (c) the molar concentration of CNH_2^- (DPP) in C_2^- (SU8). It is calculated from Counts (CNH_2^-)/[Counts (CNH_2^-) + Counts (C_2^-)].

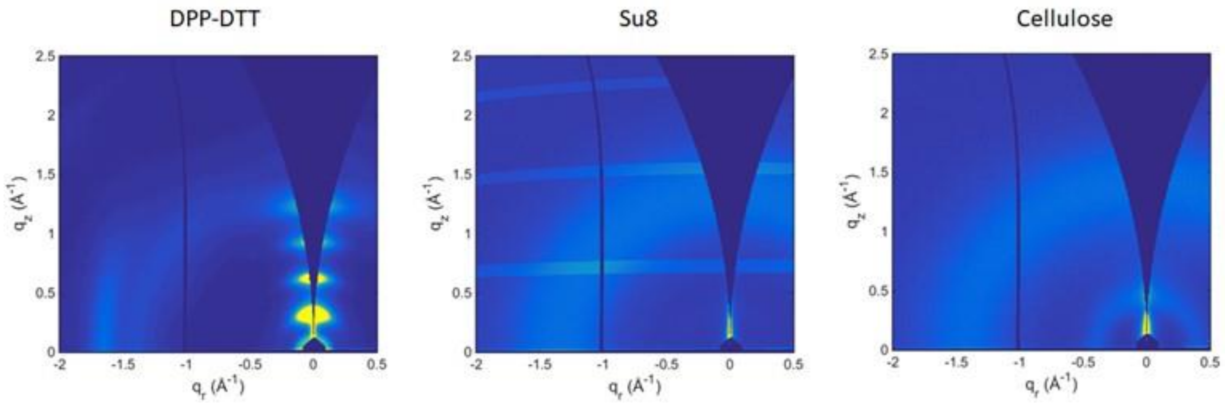

Supplementary Fig. 11. GIWAXS patterns of DPP films on OTS-treated SiO_2/Si substrate, cured SU8, and PCell film on SiO_2/Si substrates. Both the concentrations of SU8/$CHCl_3$ and PCell/$CHCl_3$ solution are 4 mg mL^{-1}. The films were annealed at 150 °C for 30 min before these images were recorded.

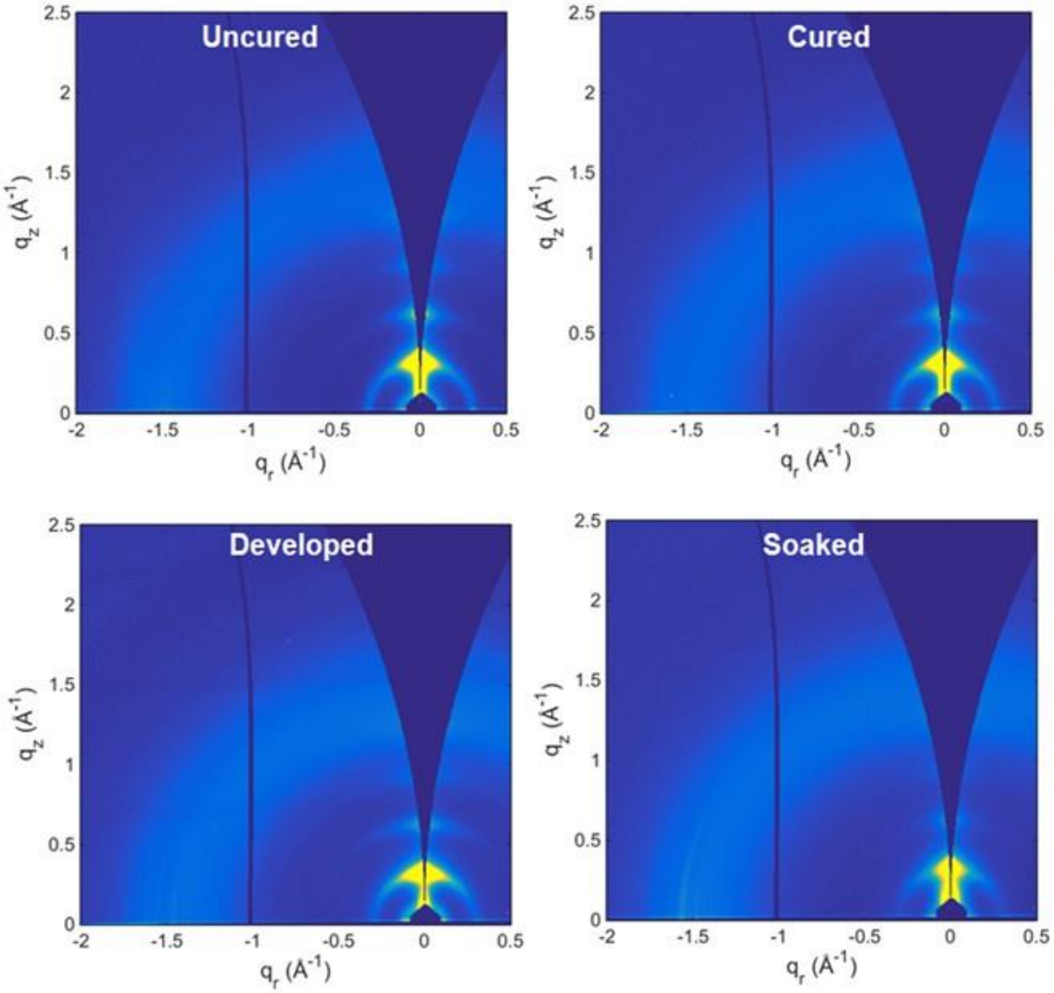

Supplementary Fig. 12. GIWAXS patterns of a 50wt% DPP/SU8 film on OTS-treated SiO_2/Si substrates after spin-coating, UV irradiation, chloroform developing and 24 h $CHCl_3$ soaking. The films were annealed at 150 °C for 30 min before these images were recorded.

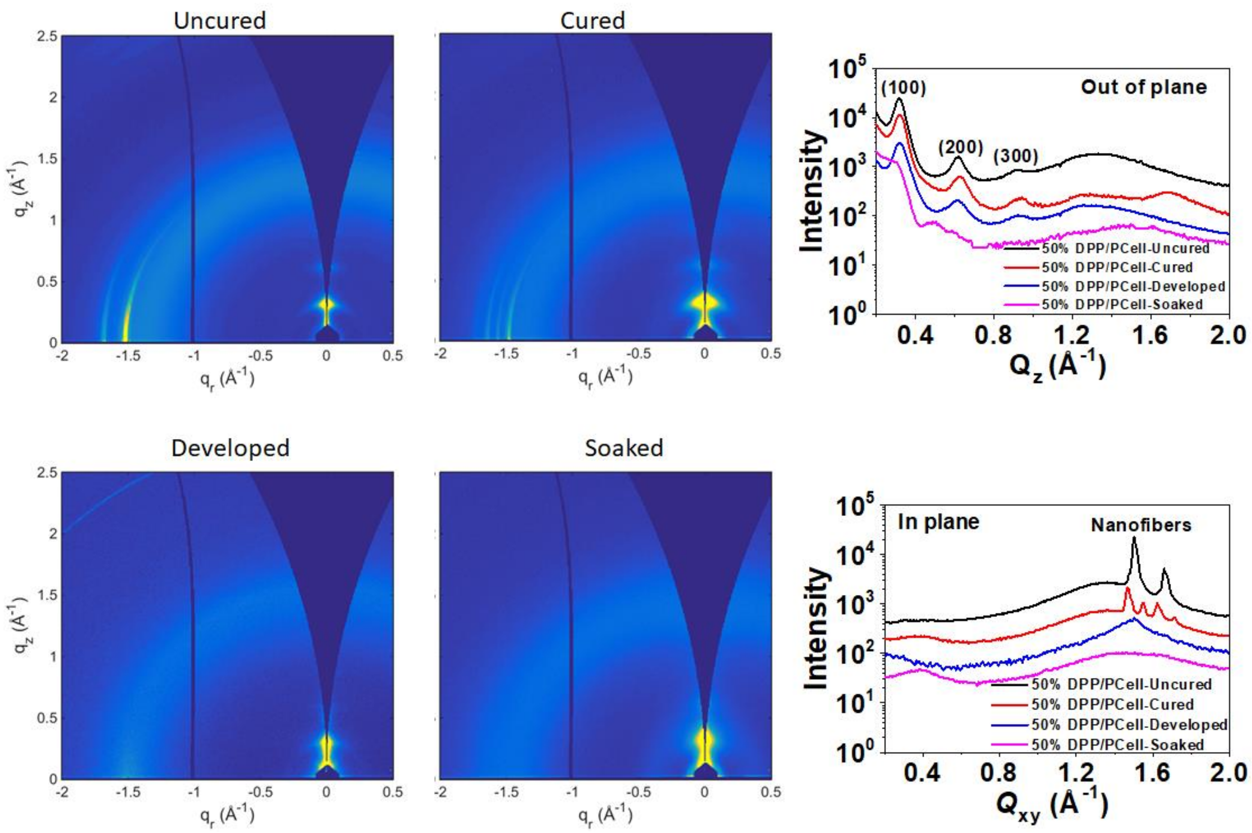

Supplementary Fig. 13. GIWAXS patterns and line cuts of 50wt% DPP/PCell films on OTS-treated SiO_2/Si substrates after spin-coating, UV irradiation, chloroform developing and 24 h $CHCl_3$ soaking. The films were annealed at 150 °C for 30 min before these images were recorded.

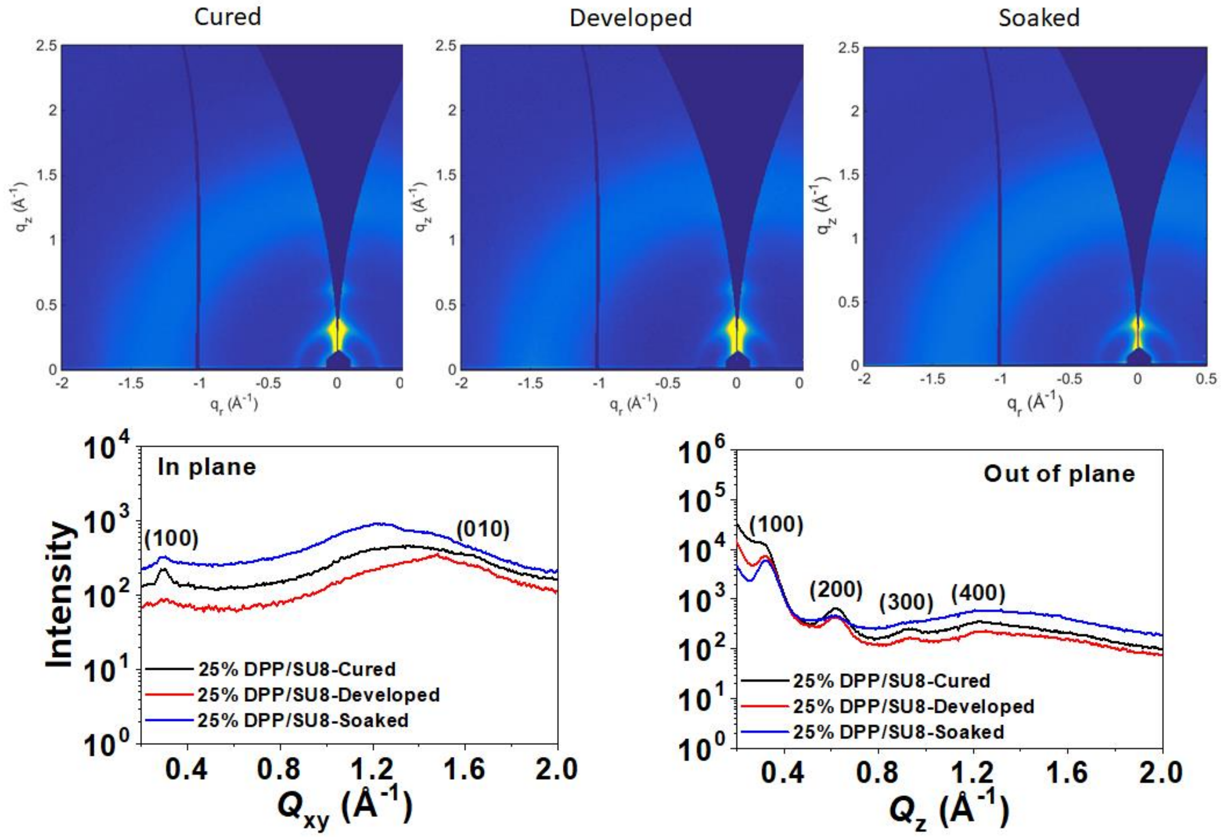

Supplementary Fig. 14. GIWAXS patterns and line cuts of 25wt% DPP/SU8 films on OTS-treated SiO_2/Si substrates after UV irradiation, chloroform developing and 24 h $CHCl_3$ soaking. The films were annealed at 150 °C for 30 min before these images were recorded.

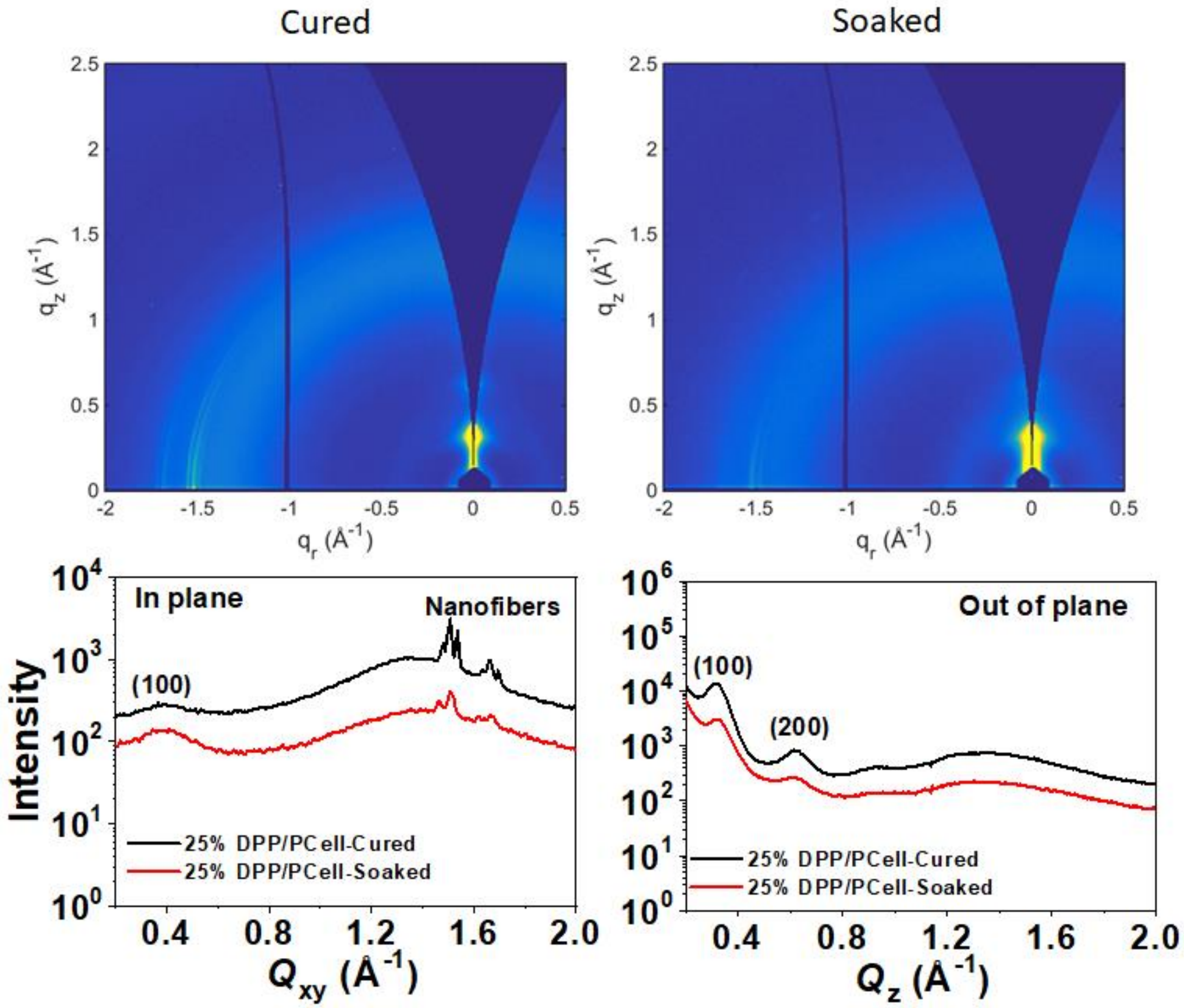

Supplementary Fig. 15. GIWAXS patterns and line-cuts of 25wt% DPP/PCell films on OTS-treated SiO_2/Si substrates after UV irradiation and 24 h $CHCl_3$ soaking. The films were annealed at 150 °C for 30 min before these images were recorded.

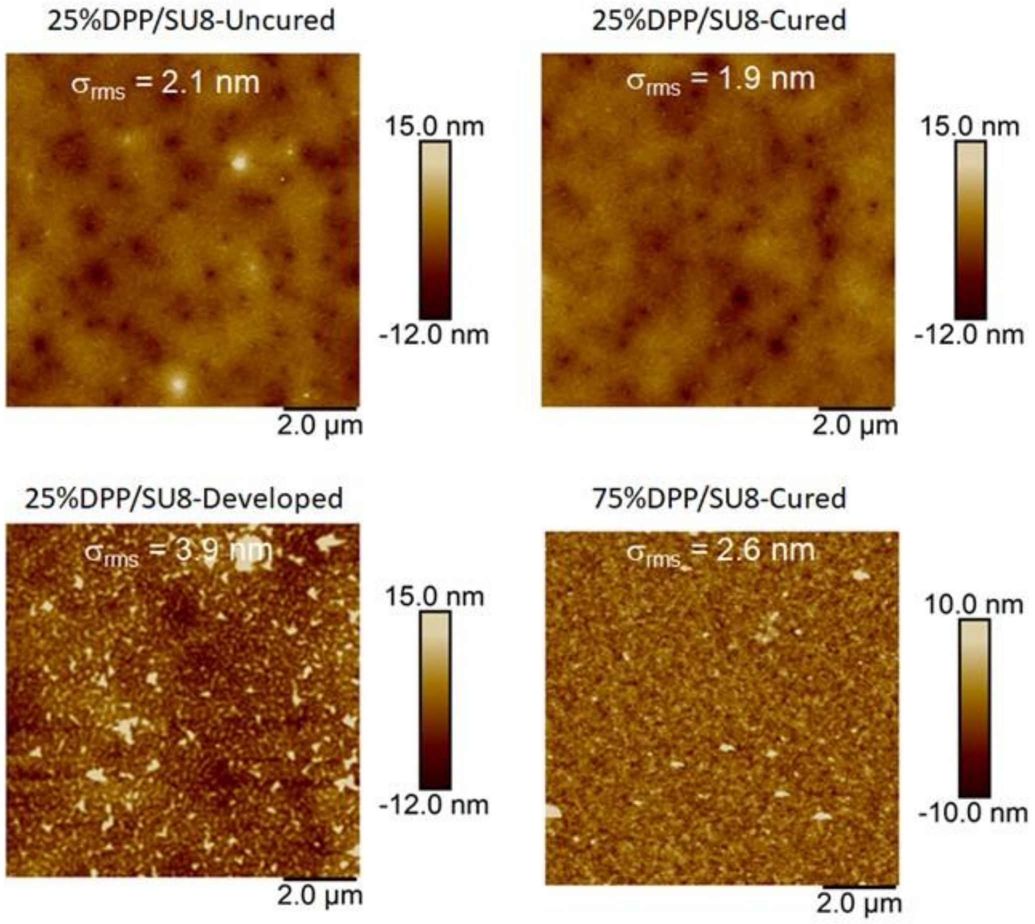

Supplementary Fig. 16. AFM images of 25 wt% DPP/SU8 film on OTS-treated SiO_2/Si substrates after spin-coating, UV irradiation, chloroform developing and 75 wt% DPP/SU8 film on OTS-treated SiO_2/Si substrate after UV irradiation. The films were annealed at 150 °C for 30 min before these images were recorded.

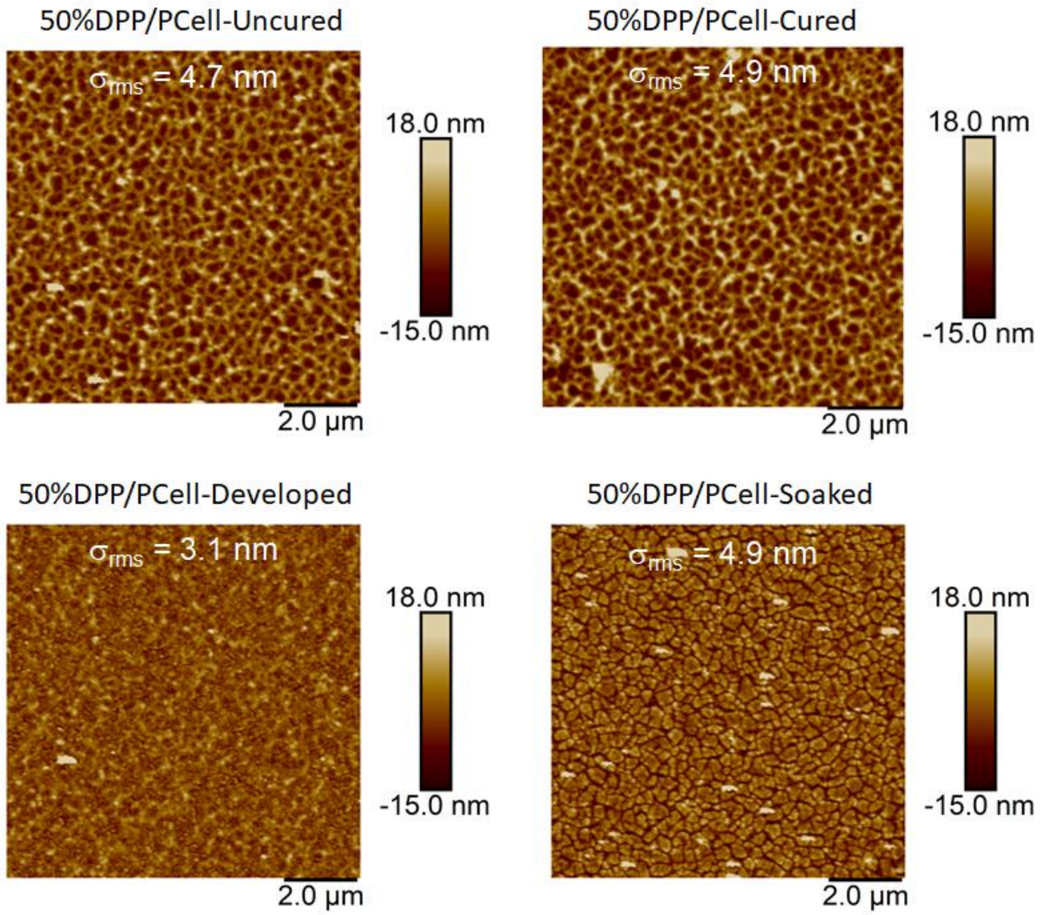

Supplementary Fig. 17. AFM images of 50 wt% DPP/PCell film on OTS-treated SiO_2/Si substrates after spin-coating UV irradiation, chloroform developing and 24 h immersing. The films were annealed at 150 °C for 30 min before these images were recorded.

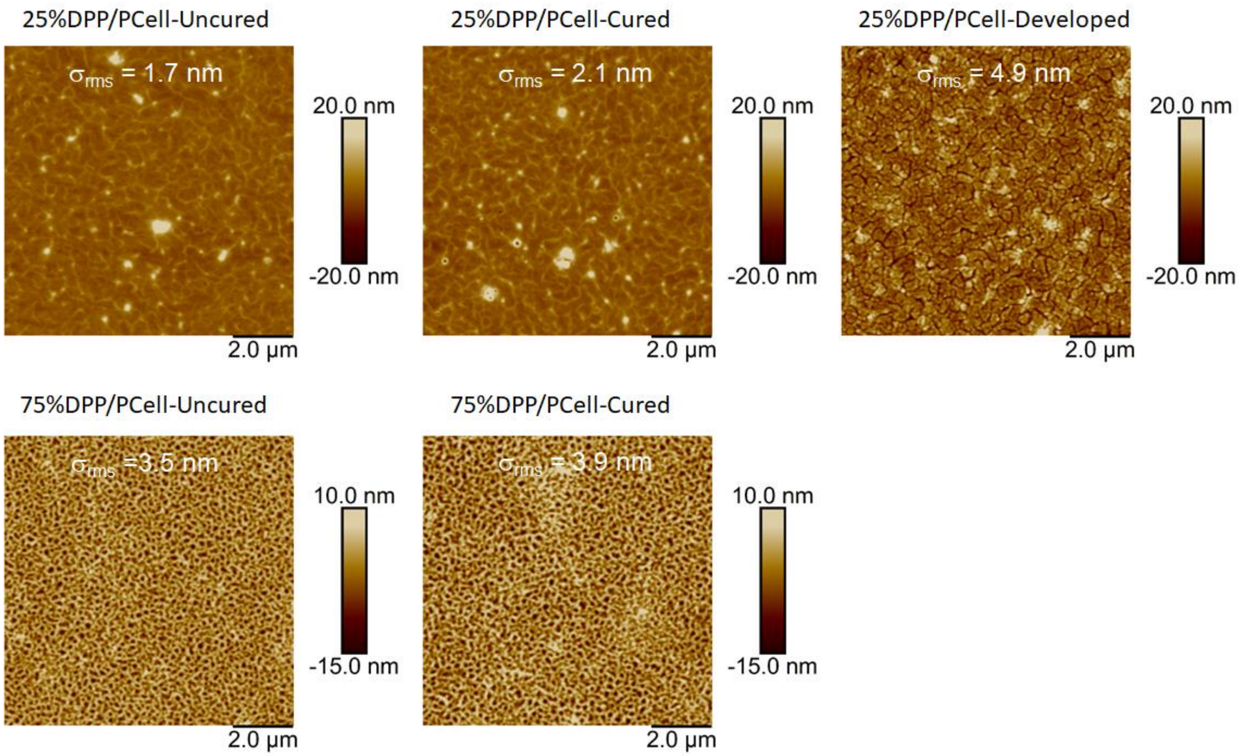

Supplementary Fig. 18. (Top) AFM images of 25 wt% DPP/PCell film on OTS-treated SiO_2/Si substrate after spin-coating, UV irradiation, chloroform developing. (Bottom) AFM image of uncured and cured 75wt% DPP/PCell film on OTS-treated SiO_2/Si substrate after spin-coating. The films were annealed at 150 °C for 30 min before these images were recorded.

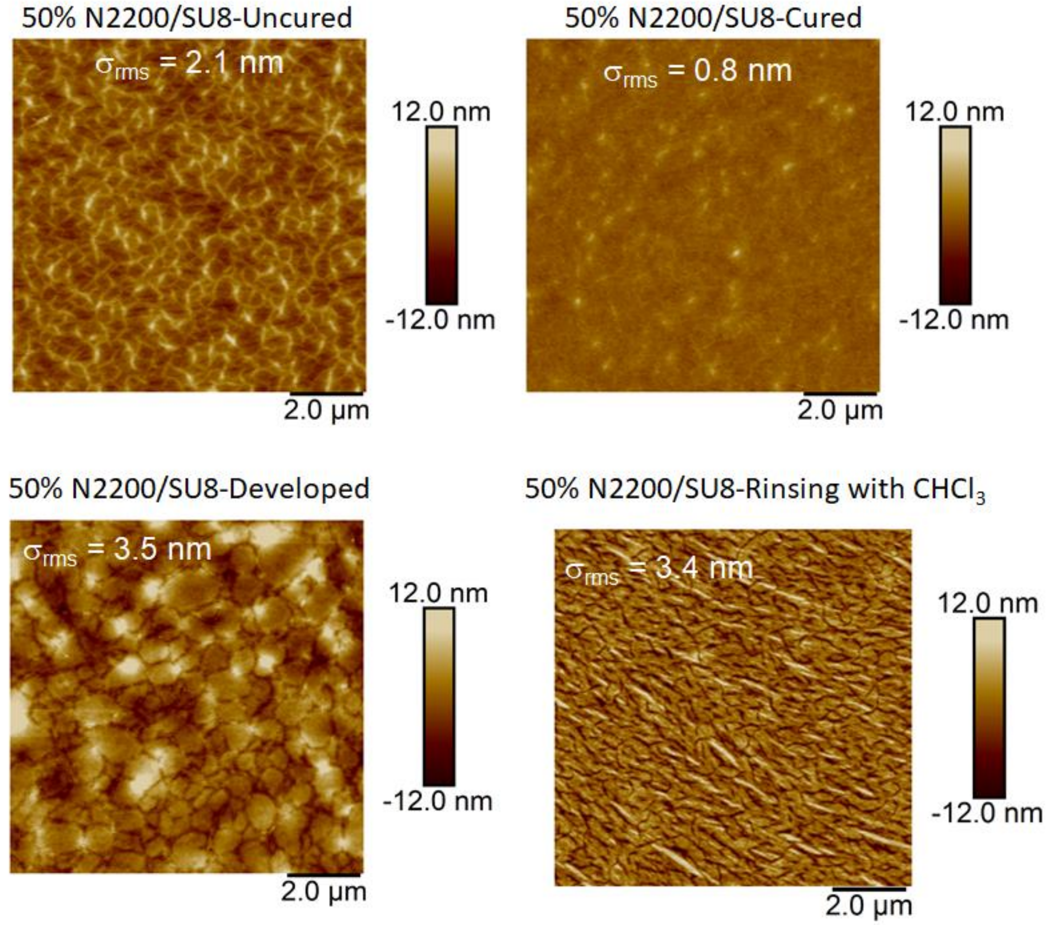

Supplementary Fig. 19. AFM images of a 50 wt% N2200/SU8 film on SiO_2/Si substrate after spin-coating, UV irradiation, SU8 developer developing, and chloroform rinsing. The films were annealed at 150 °C for 30 min before these images were recorded.

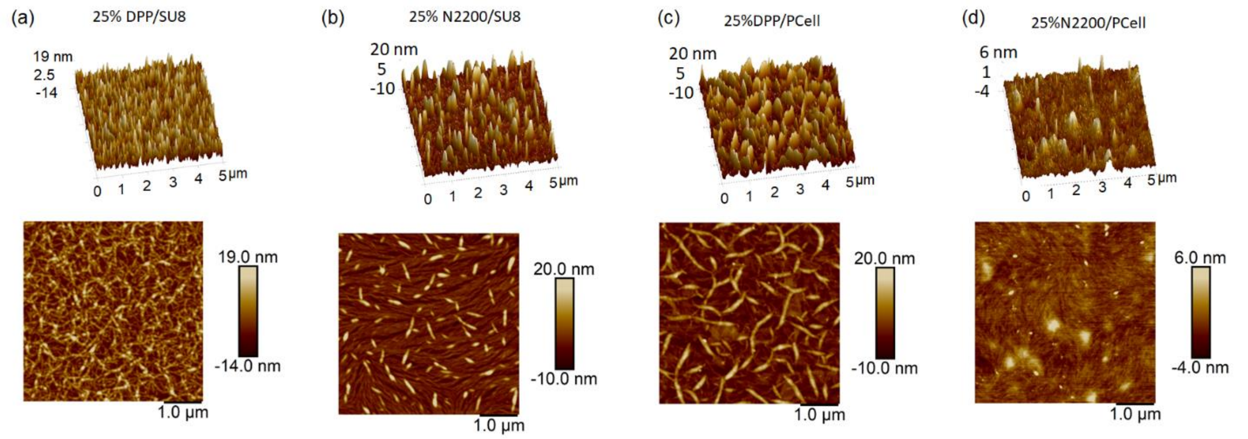

Supplementary Fig. 20. (a) 3D and 2D AFM images of DPP nanofibers after removal of SU8. (b) 3D and 2D AFM images of N2200 nanofibers after removal of SU8. (c) 3D and 2D AFM images of DPP nanofibers after removal of PCell. (d) 3D and 2D AFM images of N2200 nanofibers after removal of PCell. The average fiber diameter of DPP or N2200 is~100 nm.

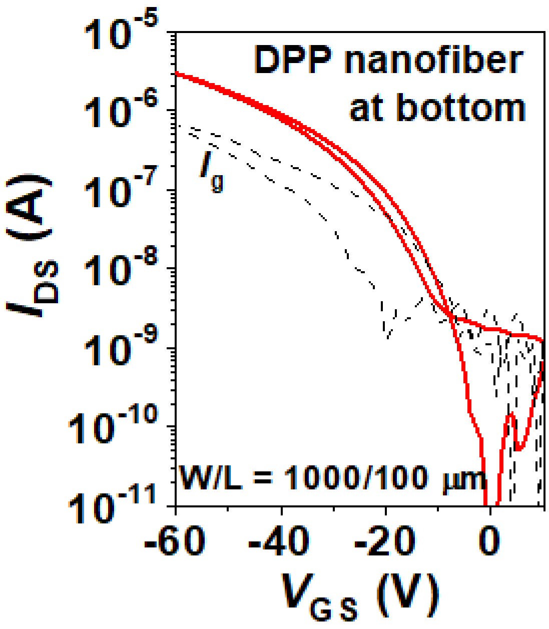

Supplementary Fig. 21. Transfer curve of BGTC OTFTs based on DPP nanofibers after removal of SU8. The calculated mobility (μ) and threshold voltage (V_t) are 0.03 cm^2 V^{-1} s^{-1} and -17.8 V, respectively.

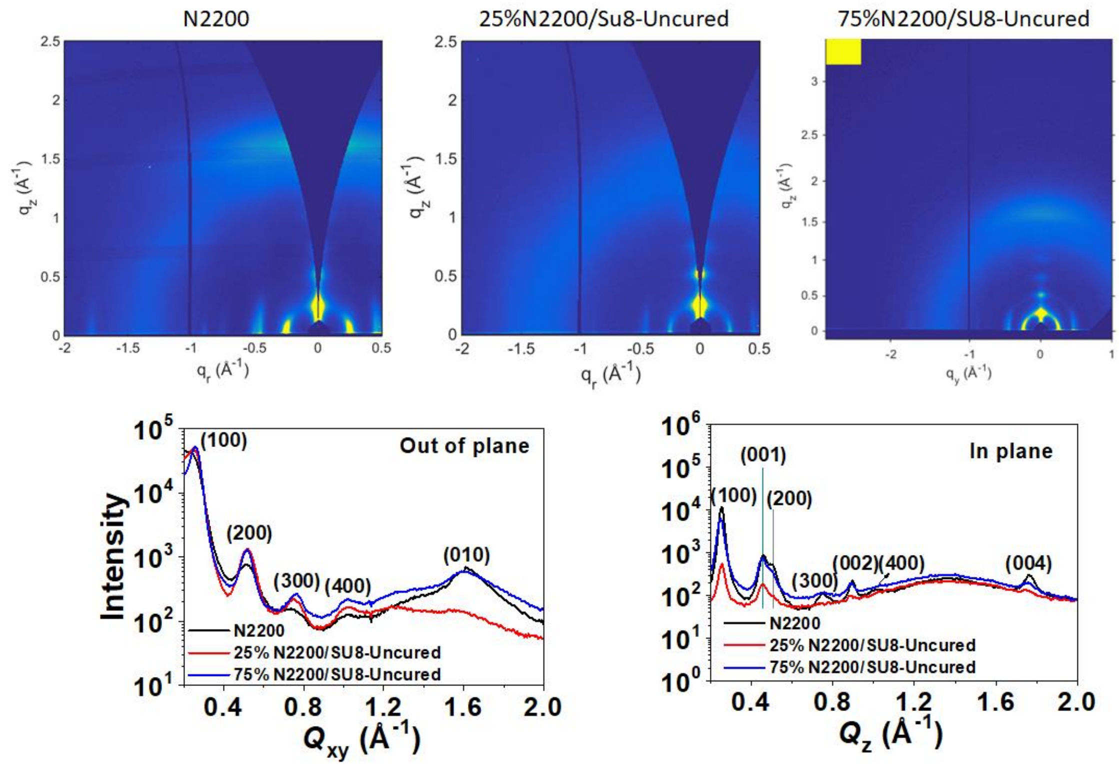

Supplementary Fig. 22. GIWAXS patterns and line cuts of spin-coated N2200, 25wt% N2200/SU8-Uncured and 75wt% N2200/SU8-Uncured films on SiO_2/Si substrates. The films were annealed at 150 °C for 30 min before these images were recorded.

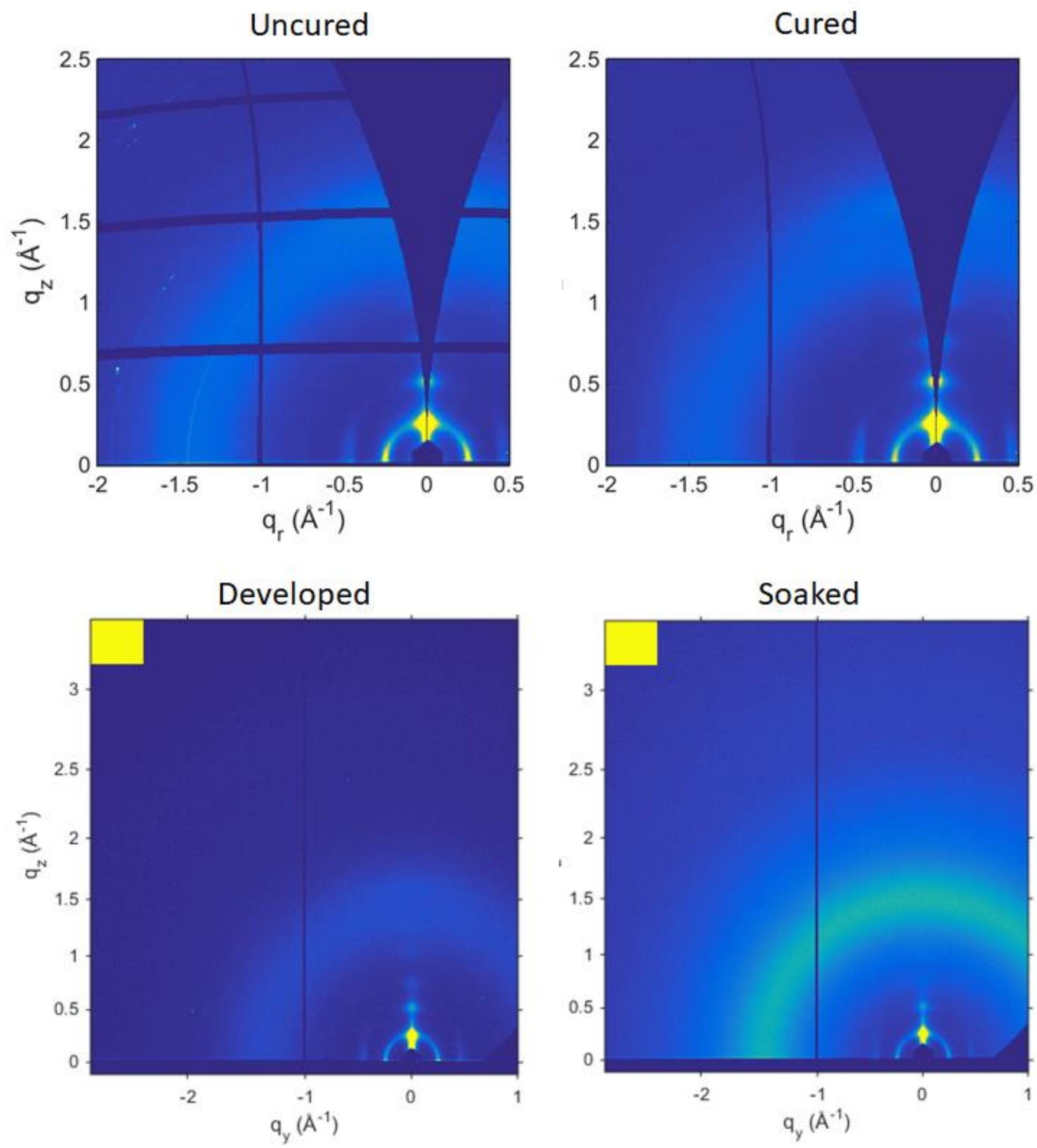

Supplementary Fig. 23. GIWAXS patterns and line cuts of 50wt% N2200/SU8 film on a SiO_2/Si substrate after spin-coating, UV irradiation, chloroform developing, and 24 h SU8 developer immersing. The films were annealed at 150 °C for 30 min before these images were recorded.

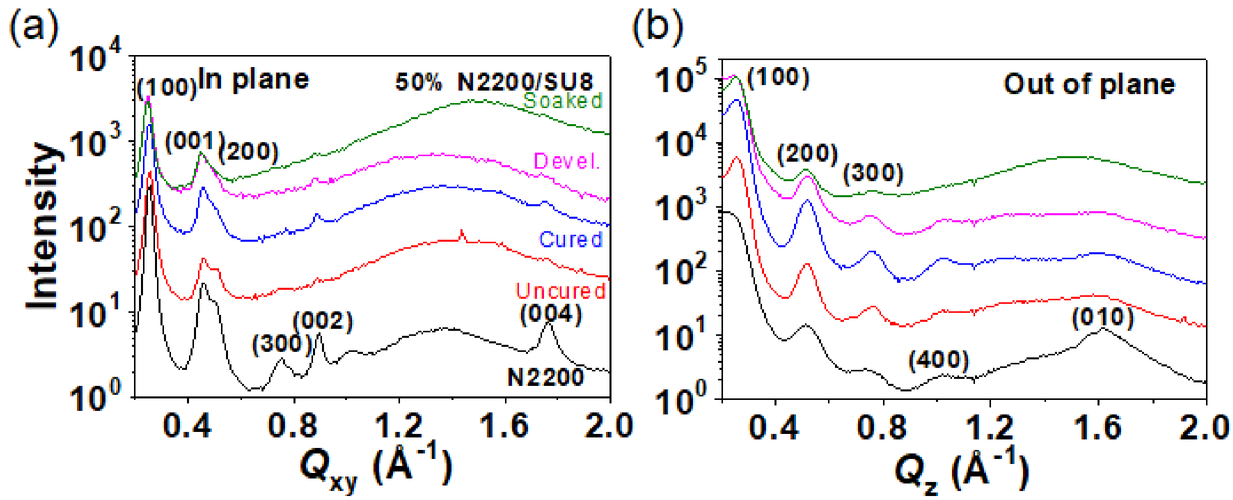

Supplementary Fig. 24. (a) In-plane and (b) out-of-plane GIWAXS line cuts of 50%N2200/SU8 films.

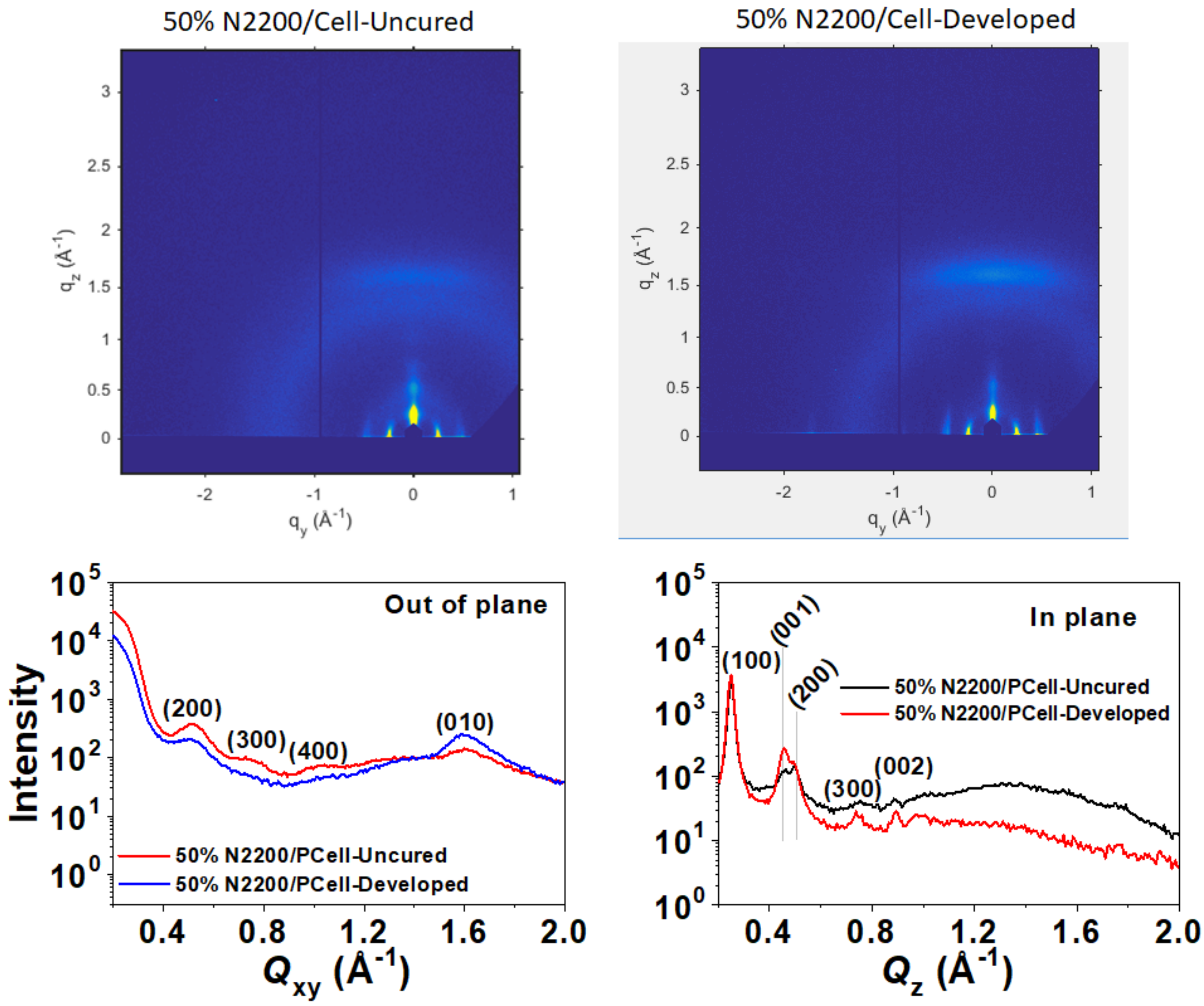

Supplementary Fig. 25. GIWAXS patterns and line cuts of a spin-coated 50wt% N2200/PCell film on SiO_2/Si substrates after spin-coating and developing by SU8 developer. The films were annealed at 150 °C for 30 min before these images were recorded.

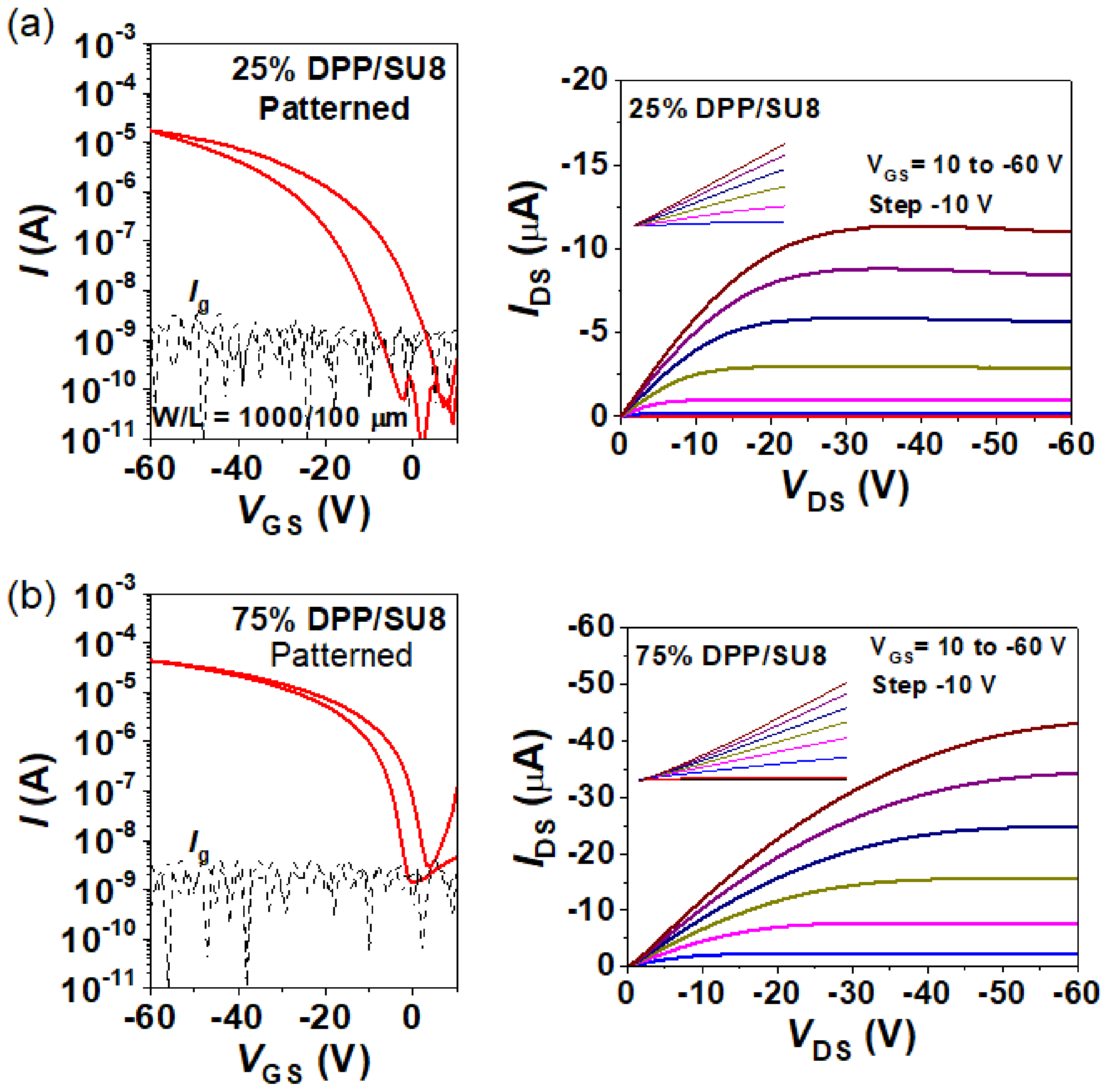

Supplementary Fig. 26. Transfer curves and output curves of BGTC OTFTs based on (a) patterned 25% DPP/SU8 and (b) patterned 75% DPP/SU8 films. The V_{DS} is -60 V for transfer curves.

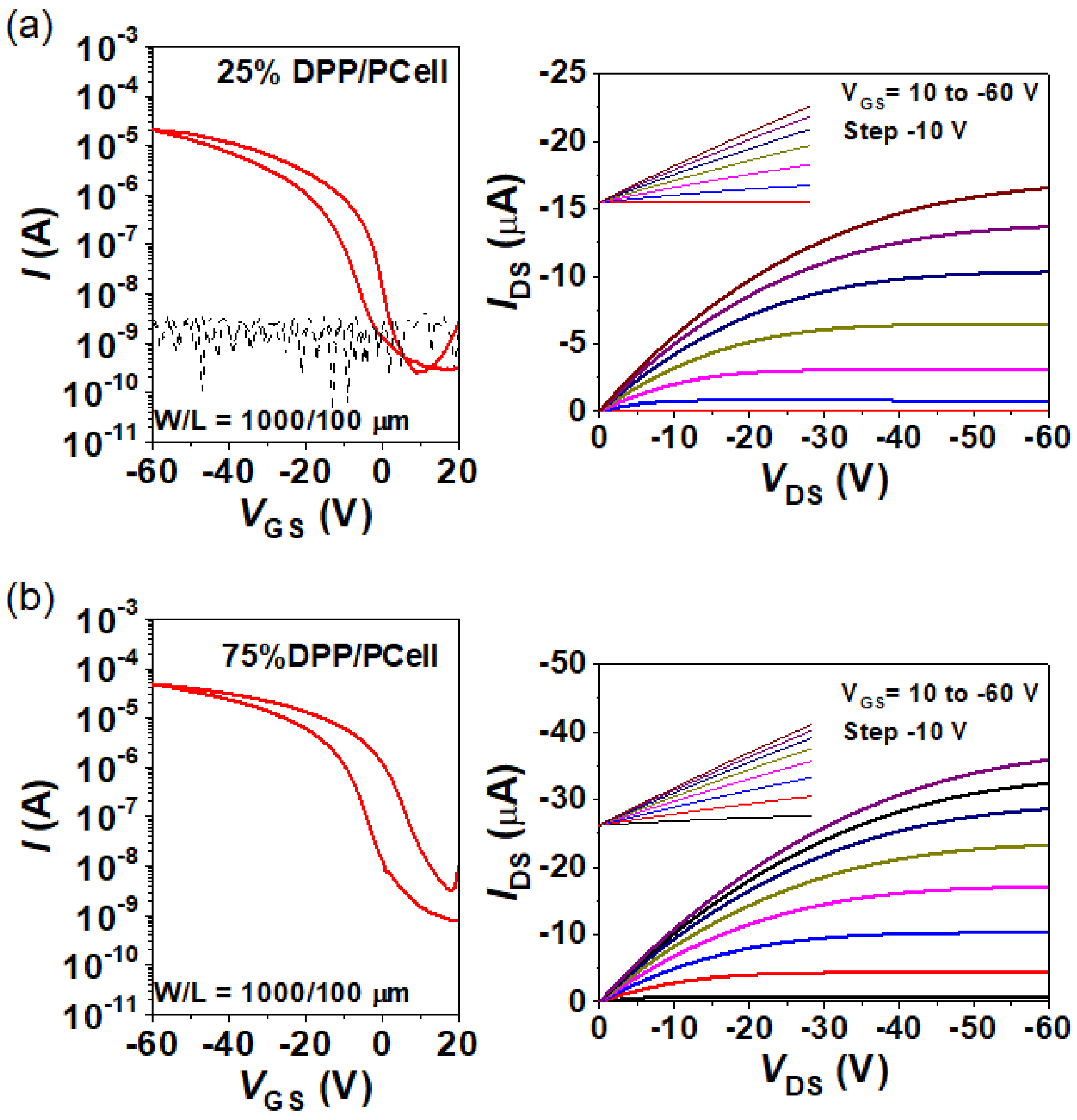

Supplementary Fig. 27. Transfer curve and output curves of BGTC OTFTs based on (a) patterned 25% DPP/PCell and (b) patterned 75% DPP/PCell films The V_{DS} is -60 V for transfer curves.

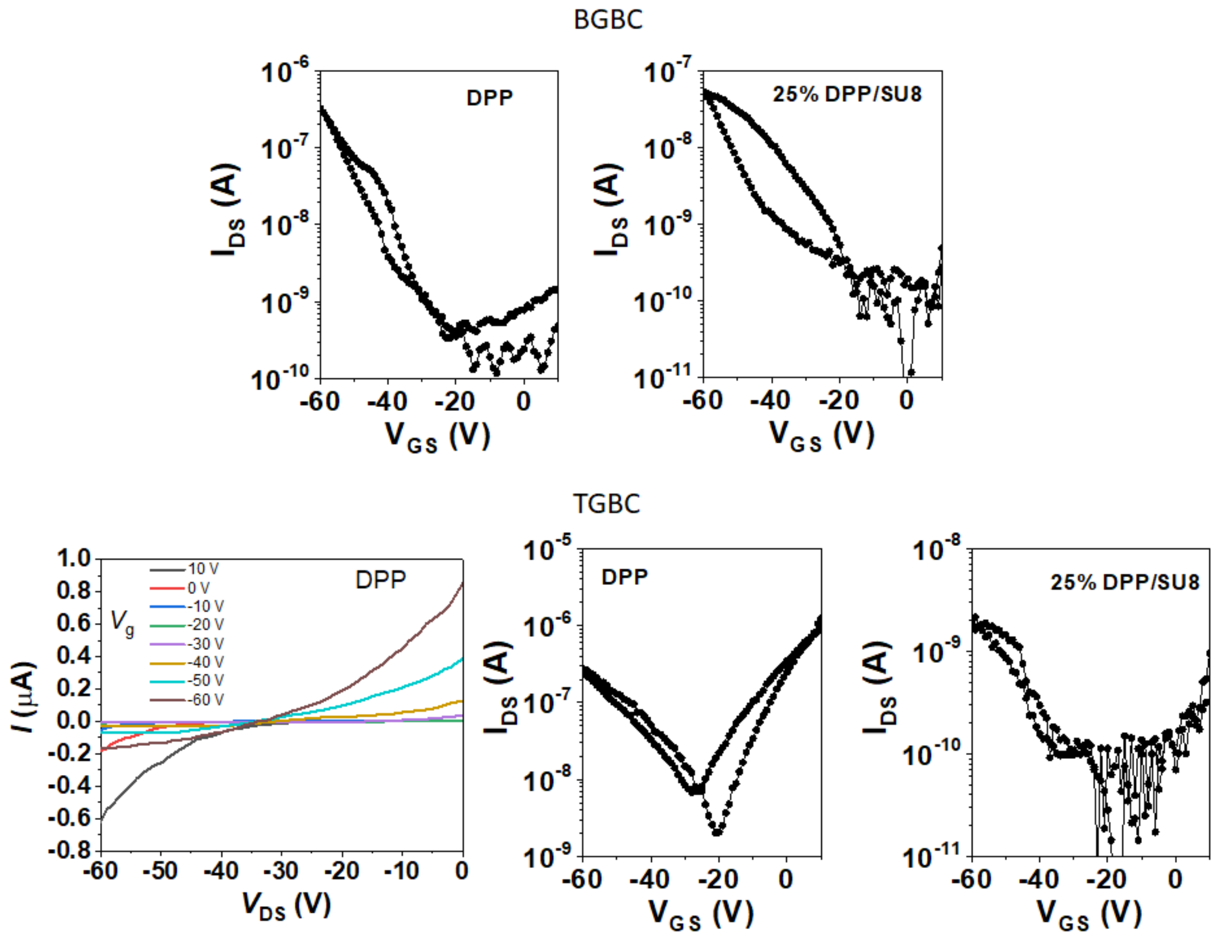

Supplementary Fig. 28. Transfer and output curves of DPP and 25%DPP/SU8 OTFTs with BGBC and TGBC structures. The low performance of devices with BGBC structure mainly originates from poor interfaces between the S/D electrodes and the semiconductor layer. The lower performance of 25%DPP/SU8 OTFTs with TGBC structure compared to DPP OTFTs originates from VPS formation with a lower DPP portion at the interface between with S/D electrodes and the semiconductor layer.

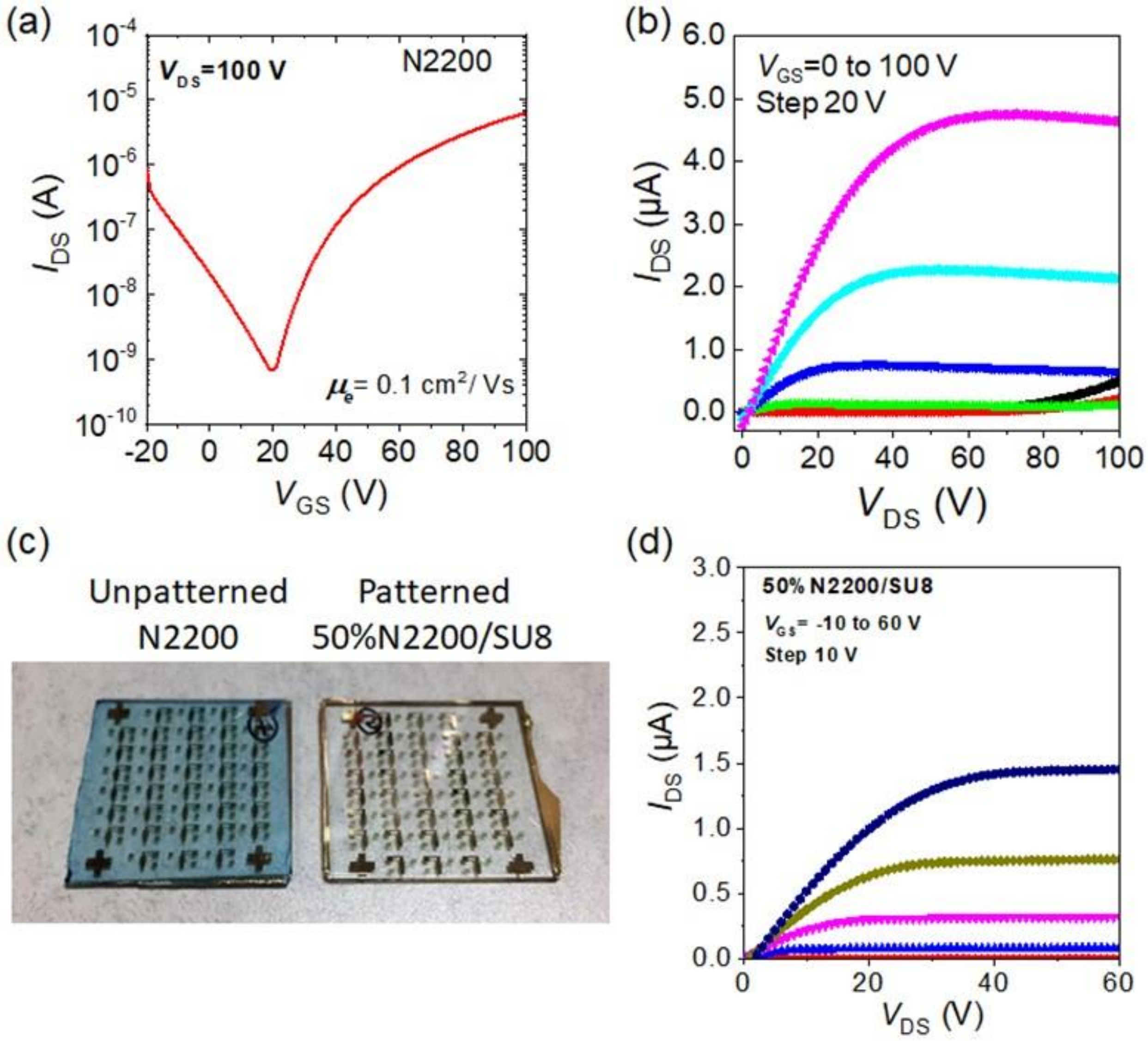

Supplementary Fig. 29. (a) Transfer curve and (b) output curve of OTFTs based on unpatterned N2200, which shows a high off current and ambipolar behavior. (c) Photo of the two device arrays. (d) Output curve of OTFTs based on patterned 50%N2200/SU8 devices.

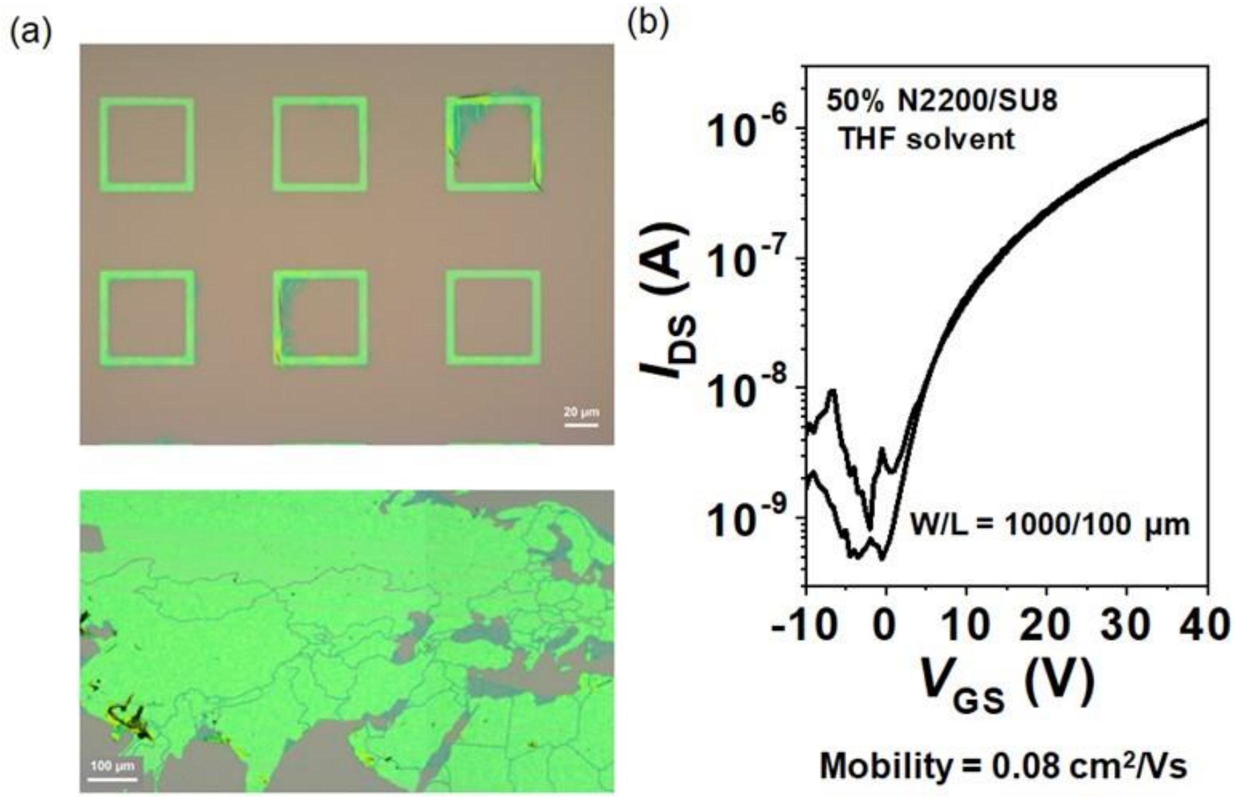

Supplementary Fig. 30. (a) Integrated optical images of patterned 50%N2200/SU8 films with tetrahydrofuran (THF) used as solvent. (b) A typical transfer curve for TGBC TFTs based on patterned 50%N2200/SU8 films.

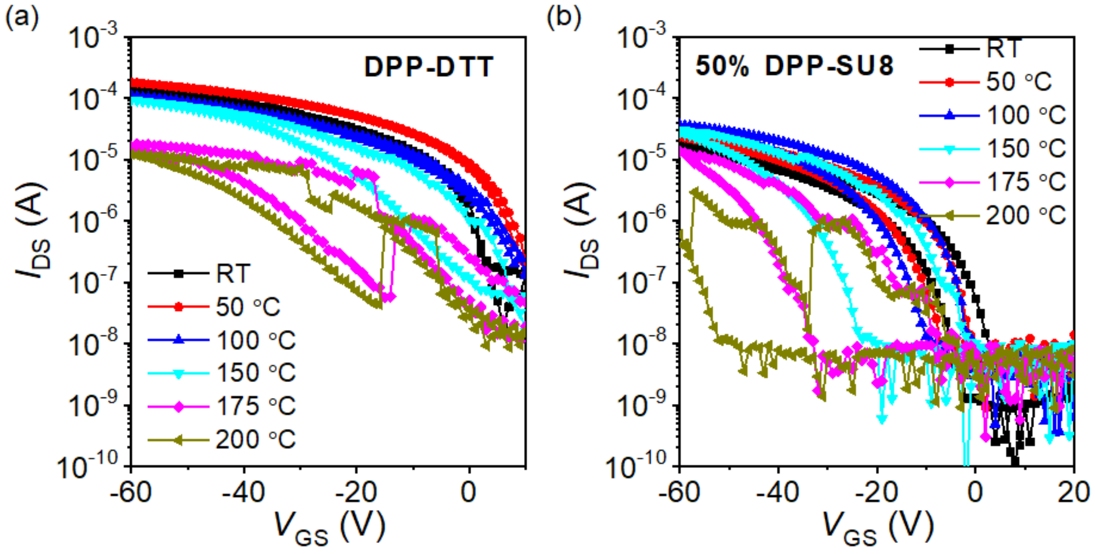

Supplementary Fig. 31. Transfer curves of a (a) BGTC DPP based OTFT and (b) BGTC 50% DPP/SU8 based OTFT at the indicated temperatures.

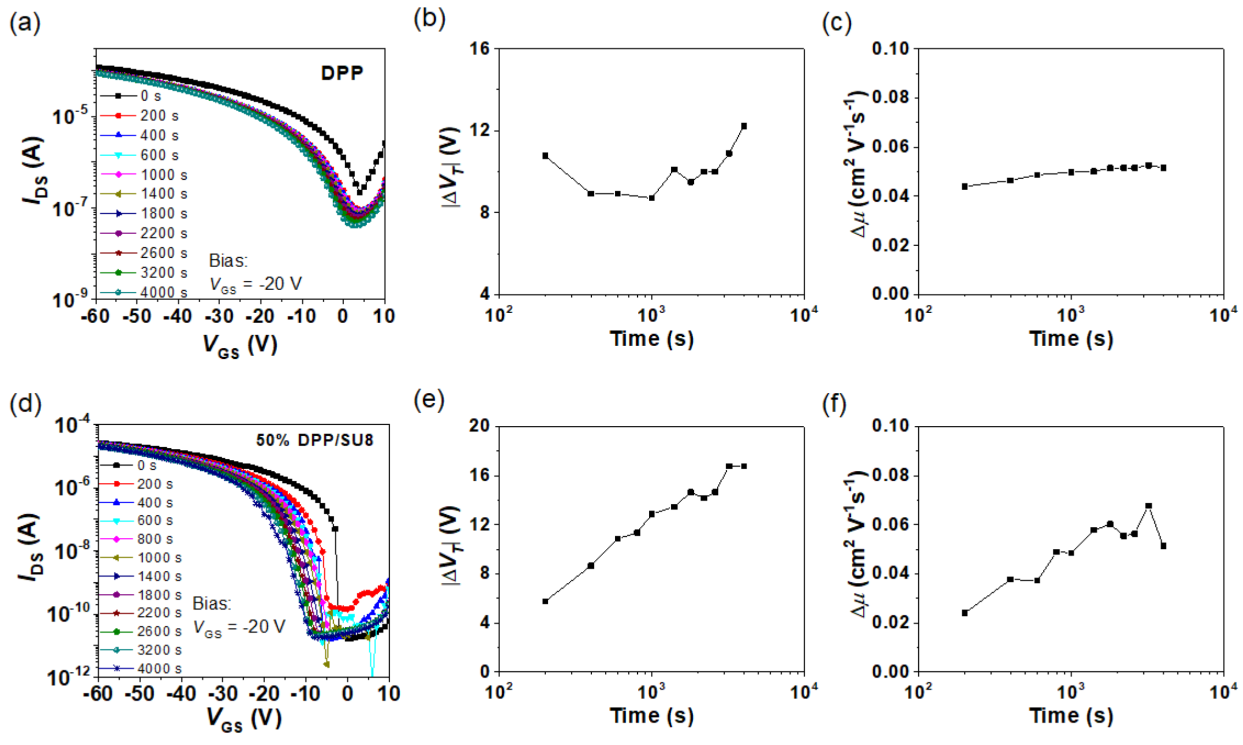

Supplementary Fig. 32. (a) The evolution of transfer curves (b) threshold voltage shift (ΔV_T) and (c) mobility shift ($\Delta\mu$) of DPP OTFTs under negative gate bias stress at a V_{GS} of -20 V for up to 4000 s. (d) The evolution of transfer curves (e) threshold voltage shift (ΔV_T) and (f) mobility shift ($\Delta\mu$) of patterned 50%DPP/SU8 OTFTs under negative gate bias stress at a V_{GS} of -20 V for up to 4000 s.

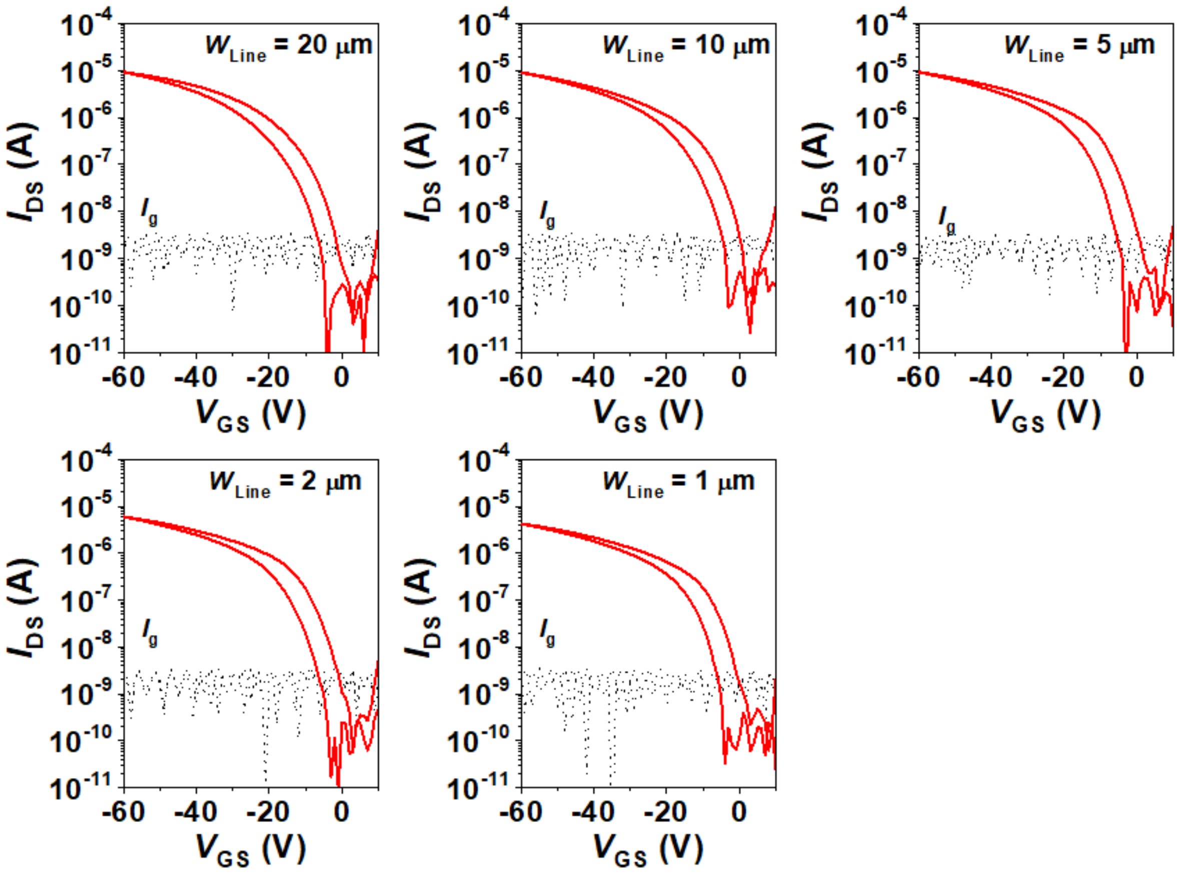

Supplementary Fig. 33. Transfer curves of BGTC 50% DPP/SU8 devices with the indicated patterned line widths. The V_{DS} is -60 V for all transfer curves.

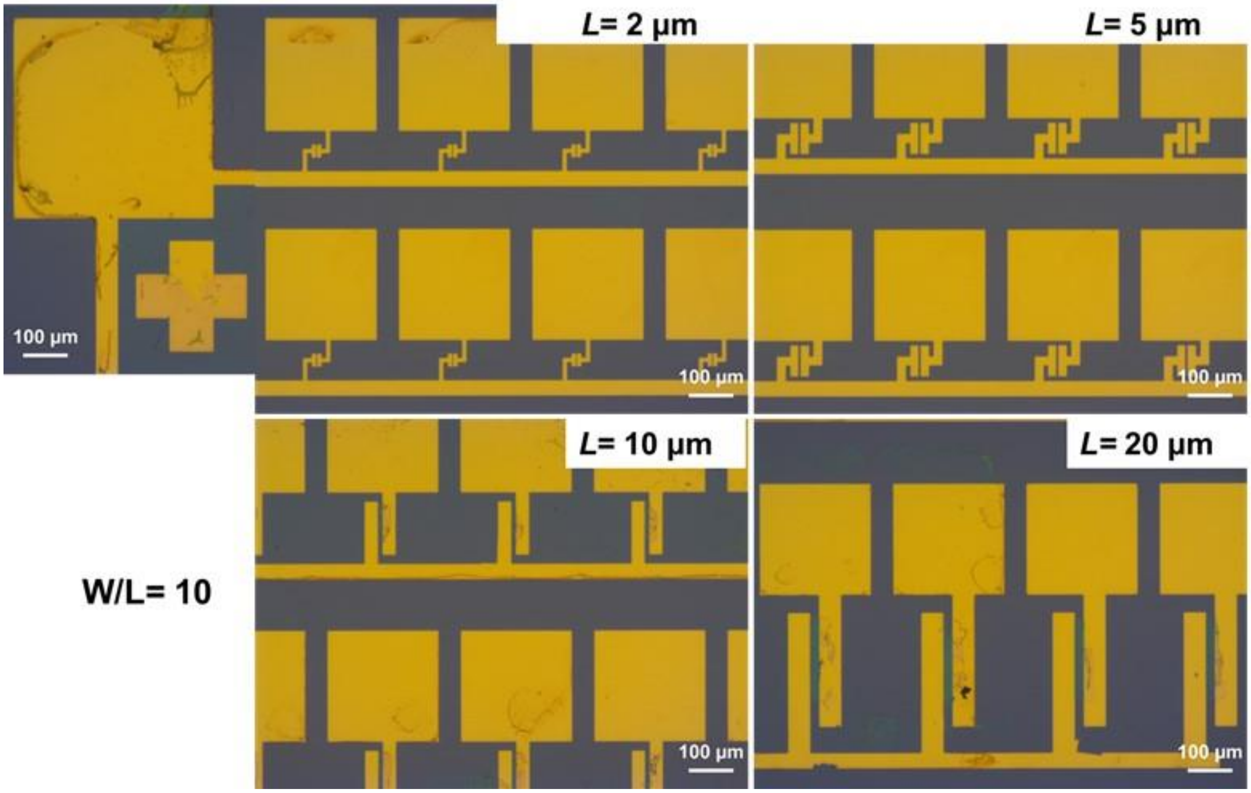

Supplementary Fig. 34. Optical images of patterned BGTC 50% DPP/SU8 device arrays with lift-off Au S/D electrodes. The ratio between the channel width (*W*) and channel length (*L*) is kept at 10.

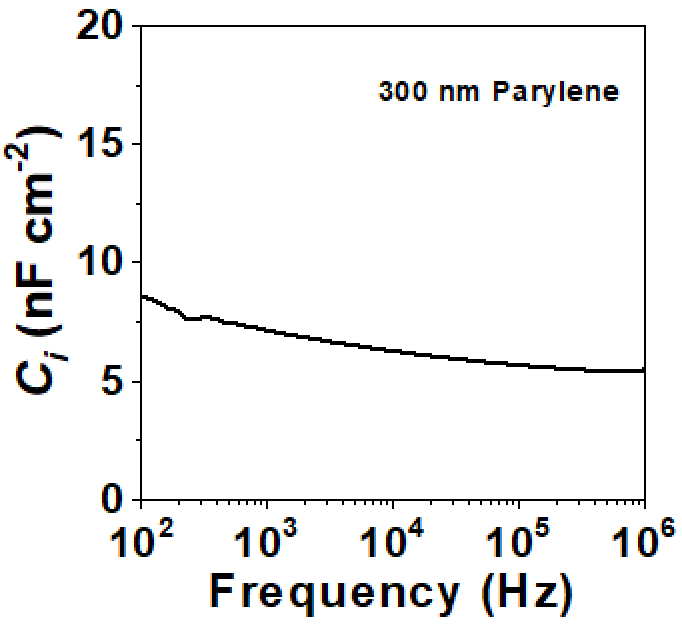

Supplementary Fig. 35. Frequency dependence of capacitance per area of 300 nm-thick parylene dielectric film.

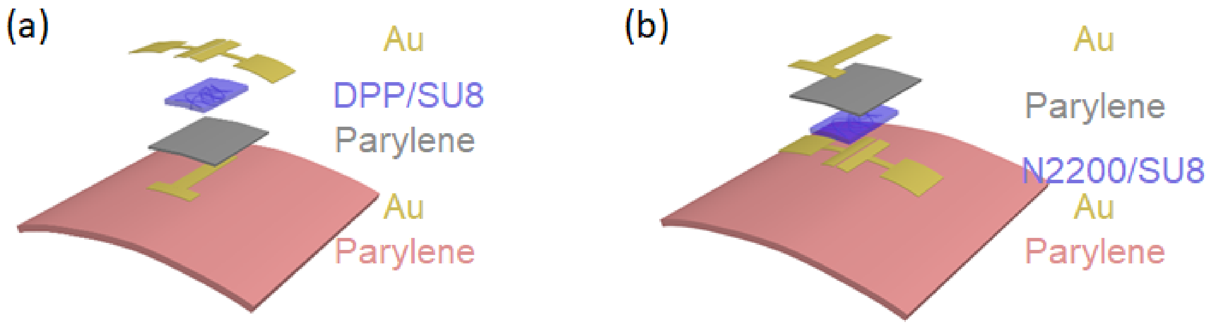

Supplementary Fig. 36. Device structure of (a) BGTC 50% DPP/SU8 and (b) TGBC 50% N2200/SU8 devices on ultraflexible parylene substrates.

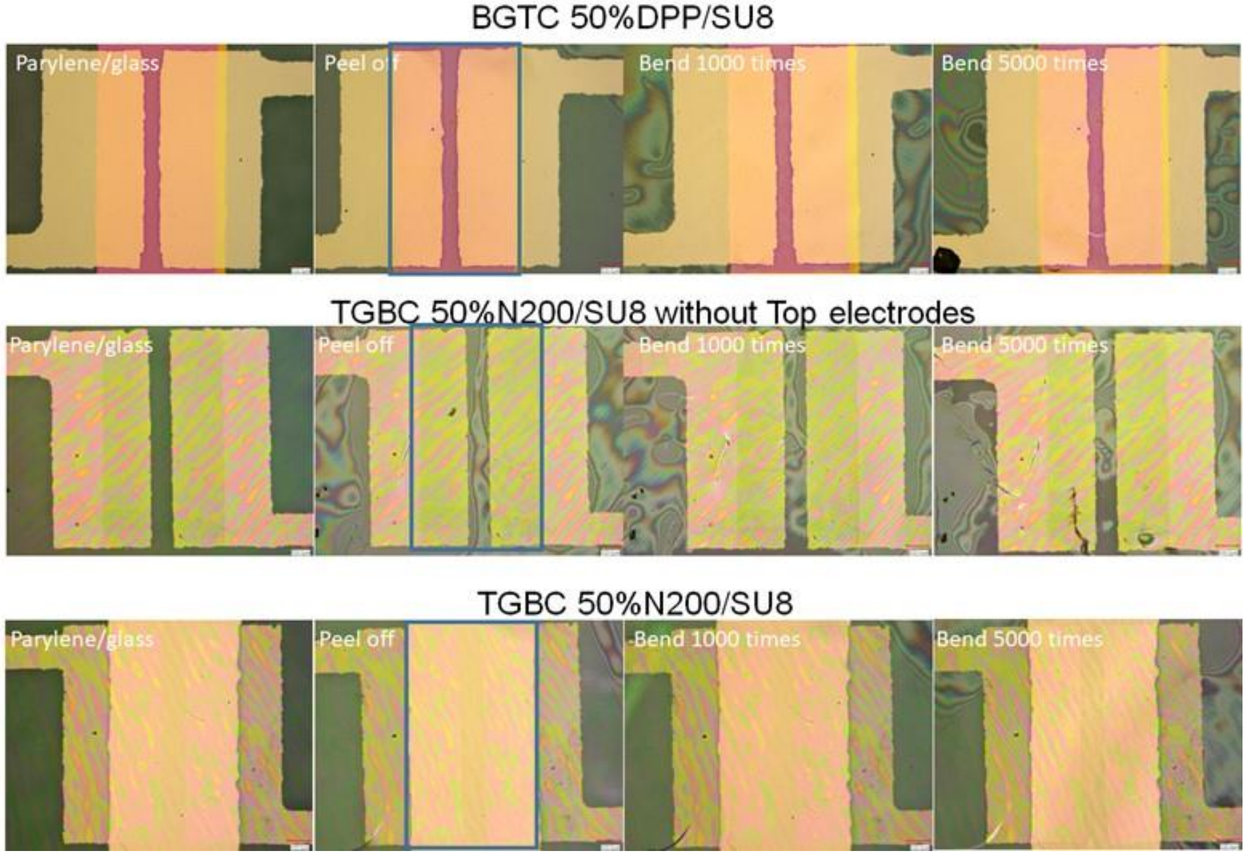

Supplementary Fig. 37. Optical images of BGTC 50% DPP/SU8, TGBC 50% N2200/SU8 OTFTs with and without top electrodes. These devices were fabricated on ultraflexible parylene polymer with glass slides as supporting substrates during device fabrication. The blue outlines show the patterned active layers.

Table S1. Summary of 2D GIWAXS parameters of the indicated films.

Film	In plane			Out of plane	
	(100)	(010)	Nanofiber	(100)	
	Q_{xy} (Å^{-1})/d (nm)	Q_{xy} (Å^{-1})/d (nm)	Q_{xy} (Å^{-1})/d (nm)	Q_z (Å^{-1})/d (nm)	ξ (nm)[a]
DPP	-/-	1.65±0.0011/ 0.38±0.0004	-/-	0.32±0.0002/ 1.97±0.003	24.6 ± 0.3
50% DPP/SU8-Uncured	0.30±0.0002/ 2.07±0.003	1.65±0.0016/ 0.38±0.0005	-/-	0.32±0.0005/ 1.97±0.005	16.8 ± 0.4
50% DPP/SU8-Cured	0.30±0.0004/ 2.13±0.005	1.65±0.0017/ 0.38±0.0003	-/-	0.32±0.0003/ 1.96±0.003	15.8 ± 0.4
50% DPP/SU8-Developed	0.30±0.0004/ 2.09±0.006	1.60±0.0019/ 0.39±0.0007	1.45±0.0012/ 0.43±0.0003	0.32±0.0004/ 1.95±0.006	17.4 ± 0.4
50% DPP/SU8-Soaked	0.30±0.0005/ 2.08±0.007	1.62±0.0014/ 0.39±0.0005	1.47±0.0016/ 0.43±0.0006	0.32±0.0002/ 1.93±0.003	15.4 ± 0.5
50% DPP/PCell-Uncured	-/-	-/-	1.50±0.0014/ 0.42±0.0005	0.32±0.0006/ 1.99±0.007	19.6 ± 0.4
50% DPP/PCell-Cured	-/-	-/-	1.47±0.0018/ 0.43±0.0007	0.32±0.0005/ 1.98±0.005	16.4 ± 0.4
50% DPP/PCell-Developed	-/-	-/-	1.50±0.0015/ 0.42±0.0004	0.32±0.0008/ 1.97±0.009	13.1 ± 0.5
50% DPP/PCell-Soaked	-/-	-/-	-/-	0.32±0.0007/ 1.95±0.007	4.96 ± 0.5

Film	(100)		(001)	(100)	(010)
	Q_{xy} (Å^{-1})/d (nm)	ξ (nm)	Q_{xy} (Å^{-1})/d (nm)	Q_z (Å^{-1})/d (nm)	Q_z (Å^{-1})/d (nm)
N2200	0.25±0.0002/ 2.48±0.004	20.0 ± 0.4	0.46±0.0011/ 1.38±0.007	0.25±0.0003/ 2.51±0.004	1.61±0.0012/ 0.39±0.0003
50% N2200/SU8-Uncured	0.26±0.0003/ 2.47±0.004	21.4 ± 0.6	0.46±0.0012/ 1.37±0.008	0.25±0.0002/ 2.51±0.007	1.60±0.0015/ 0.39±0.0005
50% N2200/SU8-Cured	0.25±0.0003/ 2.48±0.005	18.7 ± 0.5	0.46±0.0013/ 1.38±0.01	0.25±0.0004/ 2.51±0.004	1.60±0.0013/ 0.39±0.0008
50% N2200/SU8-Developed	0.25±0.0005/ 2.54±0.005	17.4 ± 0.6	0.46±0.0012/ 1.37±0.008	0.25±0.0003/ 2.51±0.006	1.60±0.0013/ 0.39±0.0007
50% N2200/SU8-Soaked	0.25±0.0007/ 2.54±0.006	17.4 ± 0.7	0.46±0.0015/ 1.37±0.009	0.25±0.0006/ 2.51±0.005	1.60±0.0016/ 0.39±0.0009

50% N2200/PCell-Cured	0.25±0.0004/ 2.50±0.005	22.4 ± 0.6	0.46±0.0013/ 1.37±0.006	-/-	1.61±0.0016/ 0.39±0.0004
50% N2200/PCell-Developed	0.25±0.0006/ 2.50±0.007	22.2 ± 0.7	0.46±0.0015/ 1.37±0.009	-/-	1.60±0.0016/ 0.39±0.0008

[a]Second order reflection (~0.62 $Å^{-1}$) used for ξ calculation.

Table S2. The contributions of range derived from the intensity *vs.* χ curve of DPP and indicated DPP/SU8 and DPP/PCell films, indicating the orientation distribution of the DPP chains.

Film	Note	Contributions of range 0°-30° (edge on)	Contributions of range 30°-90° (face on)
DPP	q = 0.28-0.35	99.6 ± 0.2%	0.4 ± 0.2%
25% DPP/SU8-Uncured	q = 0.59-0.66	62.6 ± 0.4%	37.4 ± 0.4%
25% DPP/SU8-Cured		65.4 ± 0.5%	34.6 ± 0.5%
25% DPP/SU8-Soaked		55.1 ± 0.8%	44.9 ± 0.8%
50% DPP/SU8-Uncured	q = 0.28-0.35	95.4 ± 0.3%	4.6 ± 0.3%
50% DPP/SU8-Cured		94.9 ± 0.3%	5.1 ± 0.3%
50% DPP/SU8-Developed		92.0 ± 0.3%	8.0 ± 0.3%
50% DPP-SU8-Soaked		91.0 ± 0.3%	9.0 ± 0.3%
75% DPP/SU8-Cured		89.5 ± 0.4%	10.5 ± 0.4%
25% DPP/PCell-Cured		89.5 ± 0.4%	10.5 ± 0.4%
25% DPP/PCell-Soaked		87.7 ± 0.4%	12.3 ± 0.4%
50% DPP/PCell-Uncured		93.0 ± 0.3%	7.0 ± 0.3%
50% DPP/PCell-Developed		95.1 ± 0.3%	4.9 ± 0.3%
50% DPP/PCell-Soaked		90.7 ± 0.3%	9.3 ± 0.3%
75% DPP/SU8-Cured		86.3 ± 0.5%	13.7 ± 0.5%

Table S3. Critical parameters derived from out-of-plane line-cuts of 2D GIWAXS patterns for the indicated 25% DPP/SU8 and 75% DPP/SU8 films.

Film	(100)		
	Q_z (Å^{-1})	*d* (nm)	ξ (nm)[a]
25% DPP/SU8-Uncured	0.31±0.0003	2.02±0.003	14.4 ± 0.5
25% DPP/SU8 Developed	0.31±0.0002	2.00±0.004	15.0 ± 0.3
25% DPP/SU8-Soaked	0.32±0.0004	1.96±0.006	14.1 ± 0.6
75% DPP/SU8-Cured	0.31±0.0005	2.03±0.008	17.7 ± 0.5
25% DPP/PCell-Cured	0.32±0.0003	19.69±0.007	13.8 ± 0.4
25% DPP/PCell-Soaked	0.32±0.0004	19.74±0.009	8.62 ± 0.7

[a]The second order reflection (~0.62 Å^{-1}) is used for ξ calculation.

Table S4. Critical parameters derived from in-plane line-cuts of 2D GIWAXS patterns of the indicated 25% DPP/SU8 and 75% DPP/SU8 films.

Film	(100)		(010)		Nanofiber	
	Q_{xy} (Å^{-1})	*d* (nm)	Q_{xy} (Å^{-1})	*d* (nm)	Q_{xy} (Å^{-1})	*d* (nm)
25% DPP/SU8-Uncured	0.30±0.0003	2.11±0.003	-	-		
25% DPP/SU8-Developed	0.31±0.0005	2.05±0.005	-	-	1.48±0.0012	0.43±0.0005
25% DPP/SU8-Soaked	0.30±0.0004	2.08±0.004	-	-		
75% DPP/SU8-Cured	0.30±0.0003	2.10±0.003	1.65±0.0011	0.38±0.0005	1.49±0.0013	0.42±0.0005
25% DPP/PCell-Cured	-	-	1.67±0.0013	0.38±0.0007	1.51±0.0018	0.42±0.0008
25% DPP/PCell-Soaked	-	-	1.62±0.0014 / 1.68±0.0012	0.39±0.0006 / 0.38±0.0009	1.47±0.0017	0.43±0.0006

Table S5. Critical parameters derived from in-plane and out-of-plane line-cuts of 2D GIWAXS patterns of 25% N2200/SU8-Cured and 75% N2200/SU8-Cured films.

			25% N2200/SU8-Cured	75% N2200/SU8-Cured
In-Plane	(100)	Q_{xy} (Å^{-1})	0.25±0.0004	0.25±0.0003
		d (nm)	2.49±0.006	2.53±0.004
		ξ (nm)	20.5± 0.6	21.2± 0.7
	(001)	Q_{xy} (Å^{-1})	0.47±0.0014	0.45±0.0012
		d (nm)	1.35±0.0014	1.38±0.0011
Out-of-Plane	(100)	Q_{xy} (Å^{-1})	0.25±0.0005	0.255±0.003
		d (nm)	2.49±0.005	2.47±0.004
	(010)	Q_{xy} (Å^{-1})	1.61±0.0013	1.60±0.0011
		d (nm)	0.39±0.0014	0.39±0.0012

References

1. Mochi I., Vockenhuber M., Allenet T., Ekinci Y. Open-source software for SEM metrology. *Photomask Technology* **11518,** 115180G.(2020).
2. De Simone D., Vesters Y., Vandenberghe G. Photoresists in extreme ultraviolet lithography (EUVL). *Advanced Optical Technologies* **6**, 163. (2017).
3. Cutler C., Thackeray J. W., DeSisto J., Nelson J., Lee C.-B., Li M., *et al.* Roughness power spectral density as a function of resist parameters and its impact through process. In: Kye J, editor. *Proc. SPIE Optical Microlithography XXXI.* San Jose, California, United States; 2018. p. 1058707.
